# Expression of *IbVPE1* from sweet potato in Arabidopsis affects leaf development, flowering time and chlorophyll catabolism

**DOI:** 10.1186/s12870-019-1789-8

**Published:** 2019-05-06

**Authors:** Jiaojiao Jiang, Jianzhong Hu, Rujiao Tan, Yonghua Han, Zongyun Li

**Affiliations:** 1 Institute of Integrative Plant Biology, School of Life Science, Jiangsu Normal University, Xuzhou, 221116 China; 2 Department of Plant Biotechnology, College of Agriculture and Life Sciences, Chonnam National University, Gwangju, 61186 South Korea; 3 Jiangsu Key Laboratory of Phylogenomics and Comparative Genomics, Jiangsu Normal University, Xuzhou, China

**Keywords:** Vacuolar processing enzymes (VPEs), *Arabidopsis thaliana*, Sweetpotato, Leaf development, Flowering, Senescence

## Abstract

**Background:**

Since their discovery, vacuolar processing enzymes (VPEs) have consistently been investigated as programmed cell death (PCD) initiators and participants in plant development and responses to biotic or abiotic stresses, in part due to similarities with the apoptosis regulator caspase-1. However, recent studies show additional functions of VPE in tomatoes, specifically in sucrose accumulation and fruit ripening.

**Results:**

Herein, we evaluated the functions of VPE from sweetpotato, initially in expression pattern analyses of *IbVPE1* during development and senescence. Subsequently, we identified physiological functions by overexpressing *IbVPE1* in *Arabidopsis thaliana*, and showed reduced leaf sizes and numbers and early flowering, and elucidated the underlying molecular mechanisms.

**Conclusions:**

The present data demonstrate functions of the VPE gene family in development and senescence and in regulation of flowering times, leaf sizes and numbers, and senescence phenotypes in *Arabidopsis thaliana*.

**Electronic supplementary material:**

The online version of this article (10.1186/s12870-019-1789-8) contains supplementary material, which is available to authorized users.

## Background

Leaf development is controlled by complex networks of hormones, enzymes, transcription factors, and microRNA. Over the life cycles of leaves, these factors contribute to the formation of leaf shapes and control leaf sizes. These and other leaf features vary widely among species, and differences within the same plant reflect multiple developmental stages and diverse environmental conditions, and the regulatory actions of endogenous hormones and transcription factors. In *Arabidopsis* plants, the plant-specific transcription factor teosinite branched1/cycloidea/pcf (TCP) has been implicated in the control of cell proliferation and differentiation, and in the regulation of leaf development [1]. The subsequent stage is known as the floral transition, and is regulated by multiple pathways that can be affected by environmental and endogenous factors [2]. Many flowering-related genes have been identified in *Arabidopsis* plants, including *LFY*, *AP1*, *SOC1*, *CO*, *FT*, *FRI*, *FLC*, *BN1*, *BN2*, *FPA*, *FVE*, *FCA*, and *LD*. These genes regulate flowering by either controlling hormones or by directly influencing the formation of floral organs.

Plant senescence is a major and essential process for plant development, and leaf senescence is a visible indictor of plant aging. The related cessation of photosynthesis and degradation of chlorophyll are regulated by many external and endogenous factors, including environmental cues, hormones, ROS, transcription factors, and nutrient deficiencies [3, 4]. Chl breakdown is a visual feature of leaf senescence, and leads to loss of green color and carotenoid or anthocyanin accumulation [5, 6]. Recently, the Chl catabolism pathway has been well-characterized in higher plants, and the ensuing genes in Arabidopsis include *CLH, PAO, RCCR, PPH, SGR,* and *NYC*, which all encode key enzymes of Chl catabolism.

Vacuolar processing enzymes (VPEs) are cysteine proteinases with functions in the processing of vacuolar proteins and the maturation of seed storage proteins [7–11]. Since the discovery of VPE and its proteolytic activity toward synthetic caspase-1 substrates, VPE functions have been linked to developmental and stress-induced programmed cell death (PCD) [12]. In Arabidopsis plants, VPE are expressed in all tissues [13, 14] and genes encoding VPE proteins have been classified as vegetative type proteins such as αVPE and γVPE [15] and βVPE in embryos and seeds [16], and δVPE, which is specifically and transiently expressed in the two cell layers of the seed coat during the earliest stage of seed development [17]. VPEs play essential roles in plant developmental and senescence–PCD, and in the accumulation of storage proteins [18]. Numerous studies of vegetative VPEs have defined functions under abiotic or biotic stress conditions, such as salt, wounding, pathogen infection, treatments with hormones such as jasmonic acid [14, 19], and in cell death caused by senescence [11] or disease [20, 21]. VPEs are reported to be up-regulated during petal and leaf senescence, indicating the potential role in plant senescence [19]. Silencing of vegetative type VPEs (NtVPE1) represses TMV-meditated hypersensitive cell death [21], and in Arabidopsis plants, VPE is reportedly involved in both fumonisin-induced cell death and developmental cell death of seed integuments [17]. Taken together, these studies demonstrate that VPEs are essential for various types of cell death during plant development and senescence, and in response to various stresses.

Numerous studies of VPEs reveal functions in PCD-related biological processes, including those related to development and stress responses. However, little is known about PCD-unrelated functions of VPE during plant growth. In the present study, we elucidated previously unknown roles of VPEs in plant development and senescence. Specifically, our data demonstrate that *IbVPE1* affects plant development and senescence by regulating TCP transcription factors and genes of AP1 and Chl catabolism pathways.

## Results

### Identification and expression patterns of *IbVPE1* in sweetpotato

**Fig. 1:**
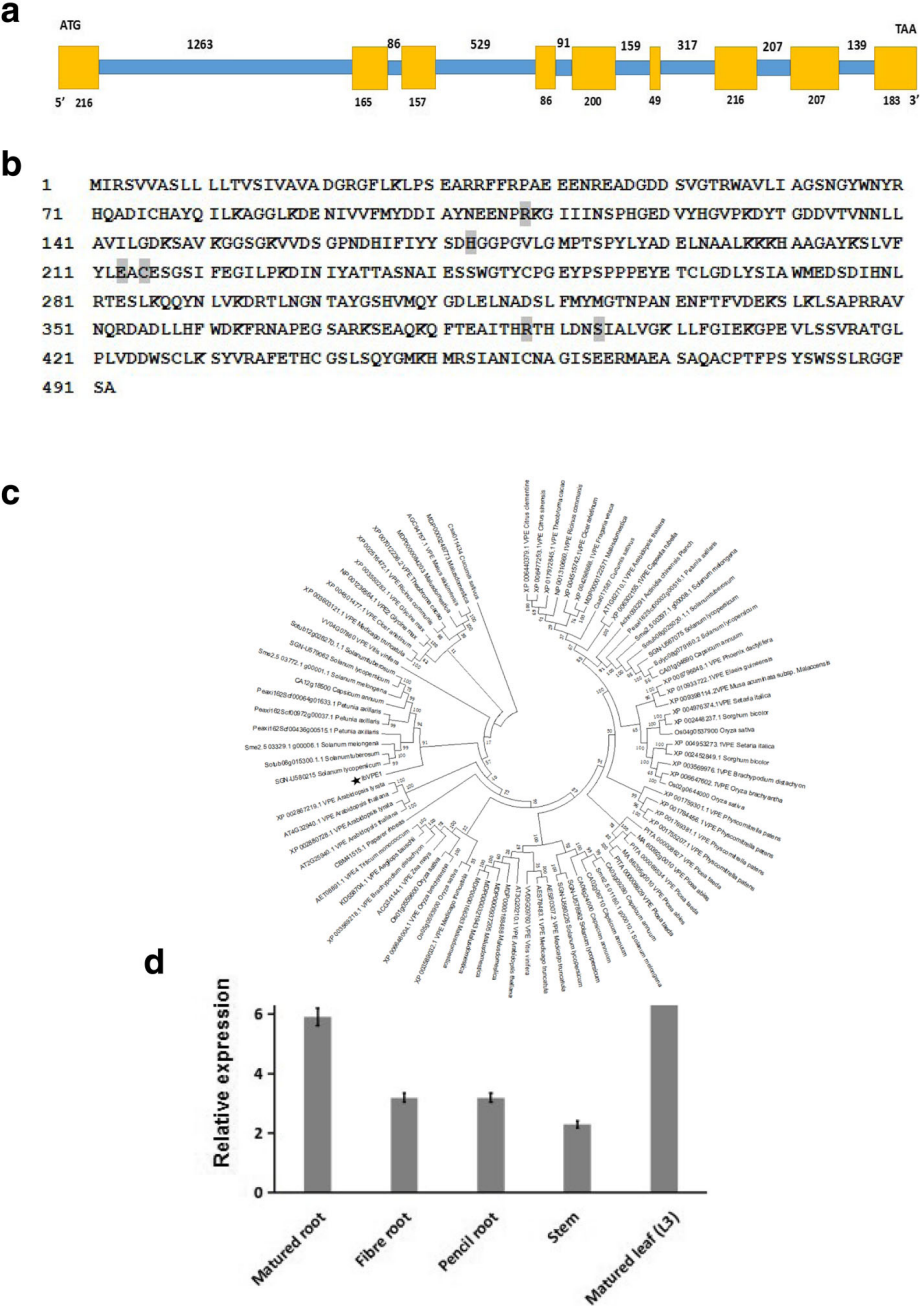
Analyses of sequences, phylogenetics, and tissue-specific expression patterns of IbVPE1; **a** *IbVPE1* gene structure including the genomic sequence and full-length cDNA; yellow boxes indicate exons and blue lines indicate introns. **b** *IbVPE1* amino acid sequence; gray boxes indicate essential amino acids for caspase-1 like activity. **c** the phylogenetic tree of *IbVPE1* was constructed from alignments with VPE proteins from other species using the neighbor-joining method in MEGA 5.0 software. **d** Tissue-specific expression pattern of *IbVPE1* in sweetpotato; *IbTublin* was used as an internal control

To determine roles of VPEs in sweetpotato, we identified and analyzed the functions of the *IbVPE1* gene by screening the Xu18 fosmid library that was constructed by the key lab of plant integrity, Jiangsu Normal University, Xuzhou, Jiangsu. After sequencing and sequence alignment analyses, full-length cDNA and genomic DNA of *IbVPE1* were identified. Comparisons of cDNA and genomic DNA revealed that *IbVPE1* contains nine exons and eight introns (Fig. 1a) and encodes a peptide of 492 amino acids. Moreover, the essential amino acids for catalytic activity and the substrate binding pocket were determined according to caspase-1 activity (Fig. 1b) [13, 22, 23]. Subsequently, we constructed a phylogenetic tree using MEGA 5.0 software and showed that the closet *AtVPE* to *IbVPE1* is *gama-AtVPE*, suggesting that it can be classified into the vegetative type of *gama-AtVPE* (Fig. 1c).

**Fig. 2:**
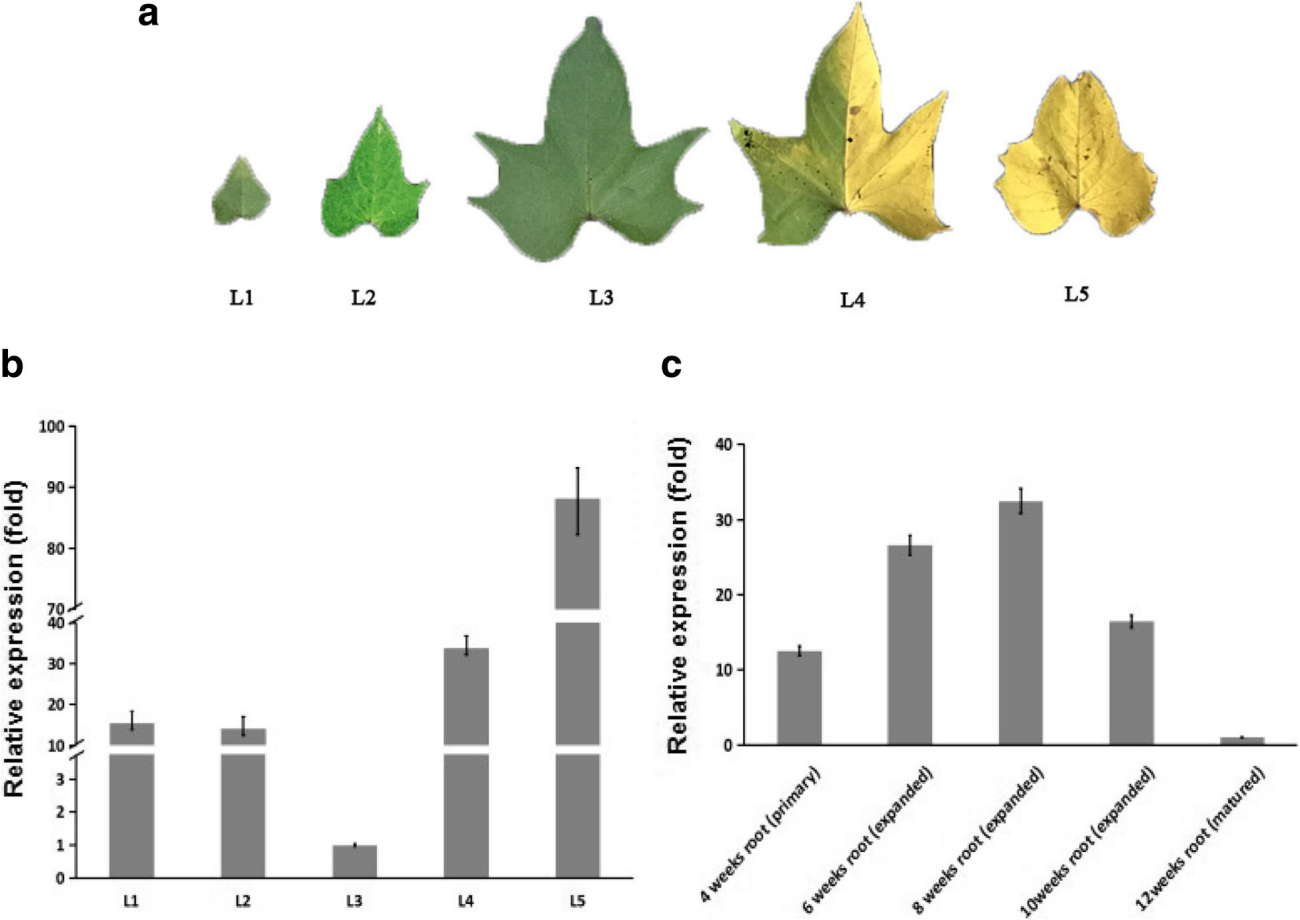
Leaf morphology and *IbVPE1* expression patterns across developmental stages of leaves and roots of sweetpotato; **a** the morphologies of sweetpotato leaves; **b** expression levels of *IbVPE1* during five developmental stages of sweetpotato leaves; **c** expression level of *IbVPE1* during five developmental stages in sweetpotato roots; *IbTublin* was used as an internal control. One-way ANOVA was used to data anaylsis

To analyze expression patterns of *IbVPE1* in sweetpotato, we examined transcription levels in various tissues and development stages using quantitative RT-PCR (qRT-PCR) and related gene expression analyses to morphologies of sweetpotato leaves (Fig. 2a). These analyses revealed that *IbVPE1* is expressed in all tissues (Fig. 2b). However, *IbVPE1* expression during leaf and root development and leaf senescence was 10–40 and 30–90 fold higher than in matured leaves and roots, respectively (Fig. 2c). These results suggest that sweetpotato *IbVPE1* is strongly transcribed during leaf and root development and during leaf senescence.

### Localization and expression pattern of *IbVPE1* in transgenic plant

**Fig. 3:**
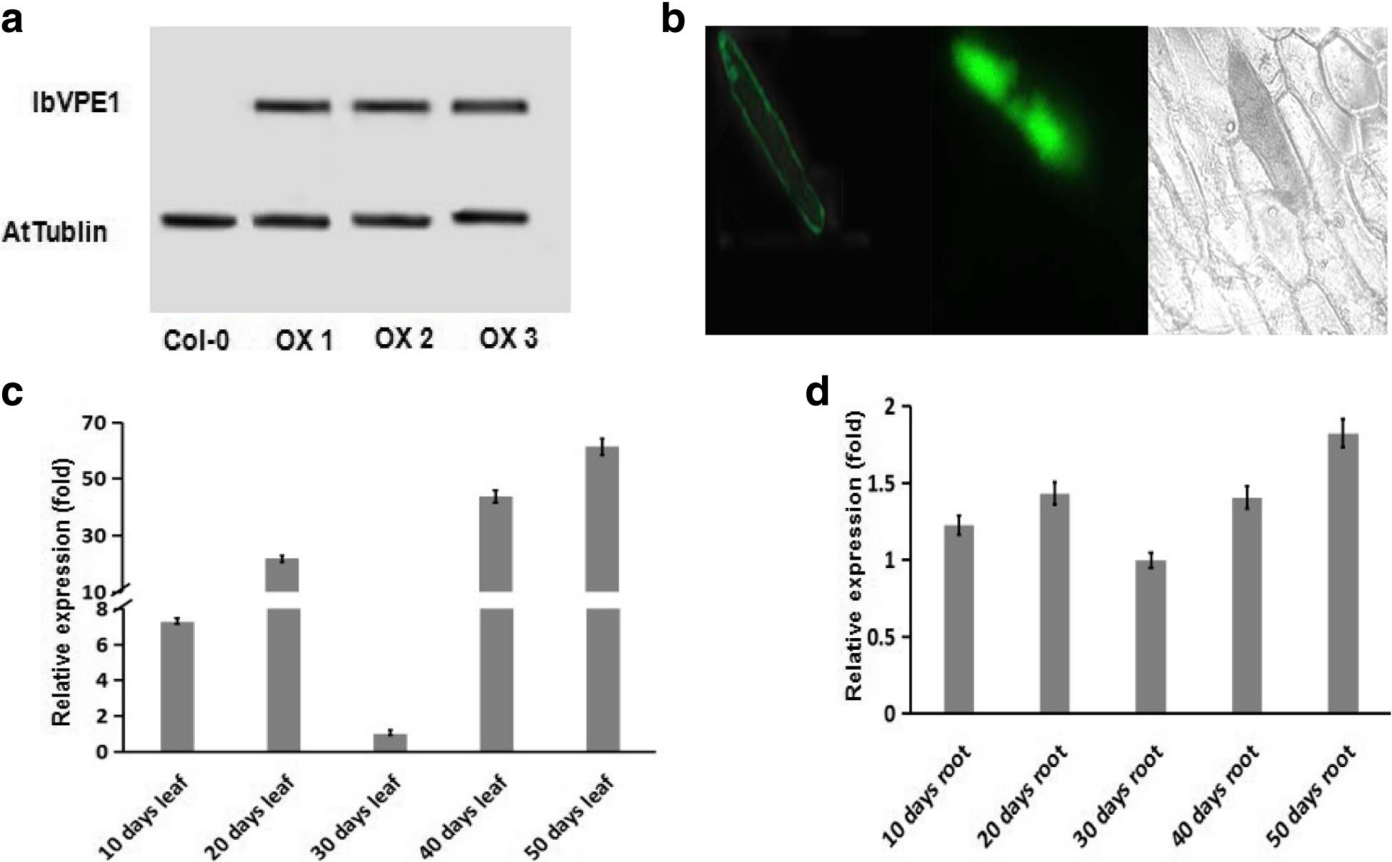
RT-PCR analysis, subcellular localization, and expression patterns of *IbVPE1*; **a** quantitative RT-PCR analyses of *IbVPE1* expression in wild-type (WT) and overexpressing (OX) plants; *AtTublin* was used as an internal control; **b** Subcellular localization of *IbVPE1* in fluorescent microscopy analyses; **c** *IbVPE1* expression levels in the five developmental stages of Arabidopsis leaves; **d** *IbVPE1* expression across the five developmental stages of Arabidopsis roots; *AtTublin* was used as an internal control. One-way ANOVA was used to data anaylsis

To study the functions of *IbVPE1*, we generated *IbVPE1* overexpressing *Arabidopsis* plants. RT-PCR analysis of homozygous T3 transgenic seedlings confirmed strong expression of *IbVPE1* in OX-1, OX-2, and OX-3 mutant seedlings compared with WT seedlings (Fig. 3a).

To confirm the subcellular localization of *IbVPE1*, full-length cDNA was fused to GFP under the control of the 35S promoter (Fig. 3b). Subsequently, we used an GFP empty vector as a negative control and stained the onion vacuolar with neutral red dye as a positive control and observed similar vacuolar localization to positive control in onion epidermis cell by fluorescence microscope.

To investigate expression patterns of *IbVPE1* in transgenic plants, we determined transcription levels in various tissues and development stages (Fig. 3c-d) by expression of *IbVPE1*-promoter-driven GUS construct in transgenic Arabidopsis plants. These experiments showed similar expression patterns to those observed in sweetpotato, including strong expression in developing and aging leaves, compared with matured leaves (30 days old leaves). However, *IbVPE1* expression levels did not change significantly during root development, suggesting an important role of *IbVPE1* during sweetpotato root tube development.

### *IbVPE1* affects leaf development and flowering times by regulating several TCP transcript factors and *AP1*

**Fig. 4:**
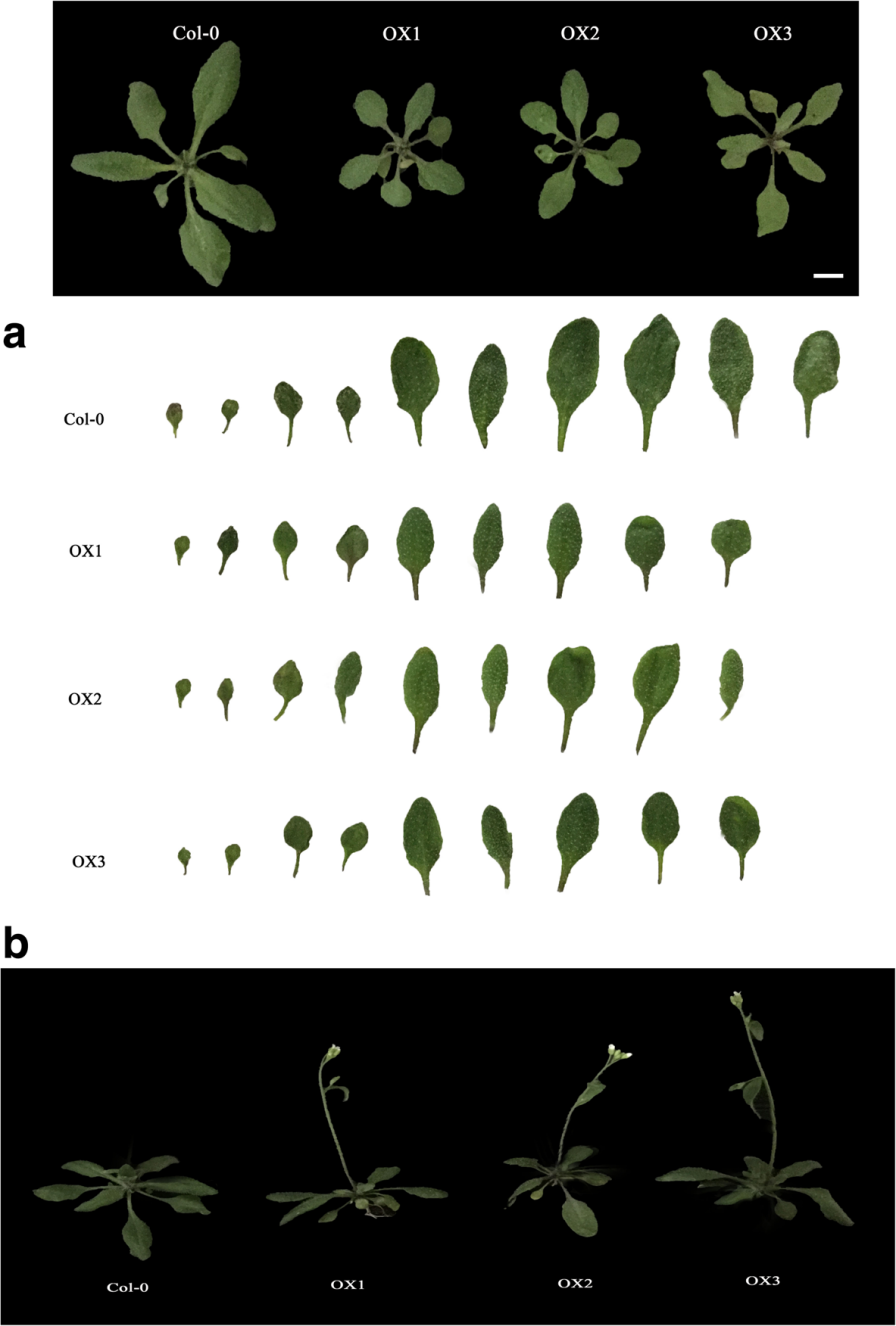
Phenotype analysis of *IbVPE1* in WT and OX plants; **a** leaf shapes and numbers in WT and OX plants at the bolting stage; **b** flowering times in WT and OX plants. The quantitative data of petiole length, blade length, blade width, blade perimeter, blade area and the number of rosette leaves are shown in Additional file 1: Figure S4

Under long-day conditions, leaf shapes of OX strains differed from those of WT plants at the bolting stage (Fig. 4a). Moreover, quantitative analyses of leaf traits showed that lengths of petioles in OX plants were similar to those in wide type plants (Additional file 1: Figure S4a), whereas OX plants had decreased blade lengths (Additional file 1: Figure S4b), blade widths (Additional file 1: Figure S4c), blade perimeters (Additional file 1: Figure S4d), and blade areas (Additional file 1: Figure S4e) compared their wild-type counterparts, and flowered comparatively early (Fig. 4b). In measurements of plant ages and rosette leaf numbers at the time of bolting, OX plants flowered an average of 5 days earlier than WT plants (Additional file 1: Figure S4f) and average numbers of rosette leaves were significantly lower than in WT plants (Additional file 1: Figure S4 g). These results suggest that *IbVPE1* affects leaf development and accelerates flowering.

**Fig. 5:**
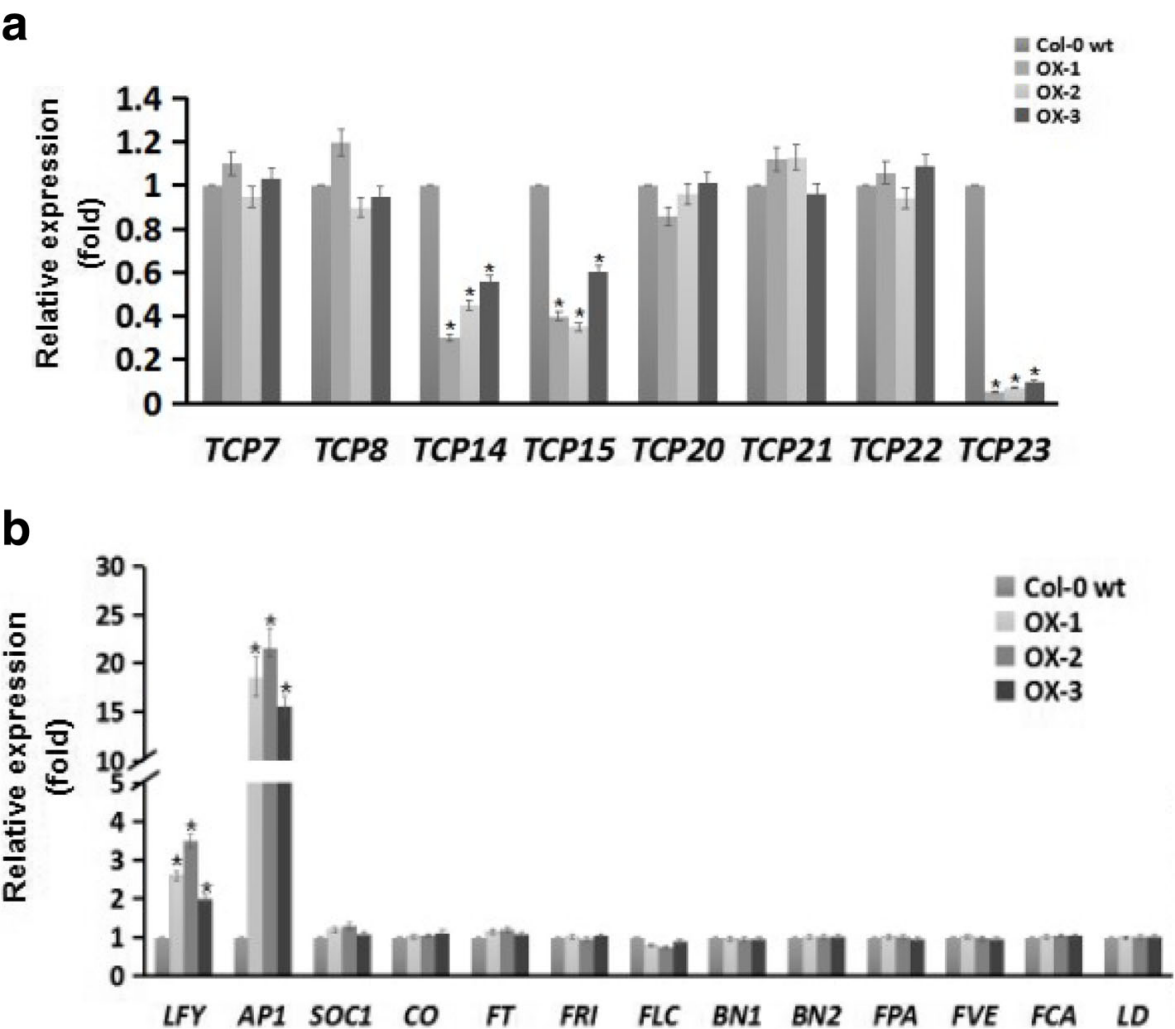
Expression levels of class I teosinte branched1/cycloidea/pcf (TCP) transcription factors and flowering-related genes; **a** *AtTCP7, AtTCP8, AtTCP14, AtTCP15, AtTCP20, AtTCP21, AtTCP22*, and *AtTCP23* expression levels in WT and OX plants; **b** *LFY*, *AP1*, *SOC1, CO, FT, FRI, FLC, BN1, BN2, FPA, FVE, FCA*, and *LD* expression levels in WT and OX plants; *AtTublin* was used as an internal control. Asterisks (*) indicate significant differences; t-test, *p* < 0.05

The TCP family of plant-specific transcription factors regulates plant phenotypes by controlling cell proliferation and differentiation, and is classified into class I and II genes. In *Arabidopsis*, class I TCP genes including *AtTCP7*, *AtTCP8*, *AtTCP14*, *AtTCP15*, *AtTCP20*, *AtTCP21*, *AtTCP22*, and *AtTCP23* reportedly regulate cell proliferation and leaf development by controlling the expression of cell-cycle genes [24–27]. Therefore, to investigate the roles of class I TCP genes in phenotypic alterations of leaves, we determined expression levels using qRT-PCR analyses. These experiments showed dramatic decreases in transcription of *AtTCP15*, *AtTCP14*, and *AtTCP23* in OX plants and slight changes in the other type-I TCP genes, compared with those in WT plants (Fig. 5a). These data suggest that *IbVPE1* affects leaf development by regulating class I TCP genes.

In addition to alterations in leaf development, we observed early flowering in OX plants compared with their WT counterparts. Thus, we determined transcript levels of genes in 4 week-old wild type and transgenic plants, which have previously been associated with flowering, including *LFY*, *AP1*, *SOC1*, *CO*, *FT*, *FRI*, *FLC*, *BN1*, *BN2*, *FPA*, *FVE*, *FCA*, and *LD*. In these gene expression analyses, *AP1* expression was significantly increased by around 20 fold and *LFY* expression was moderately increased in OX plants compared with WT plants. However, no differences in transcript levels of the other flowering-related genes were observed between OX and WT plants (Fig. 5b), indicating that *IbVPE1* primarily accelerates flowering by upregulating *AP1* expression.

### *IbVPE1* accelerates chlorophyll breakdown during dark-induced senescence

**Fig. 6:**
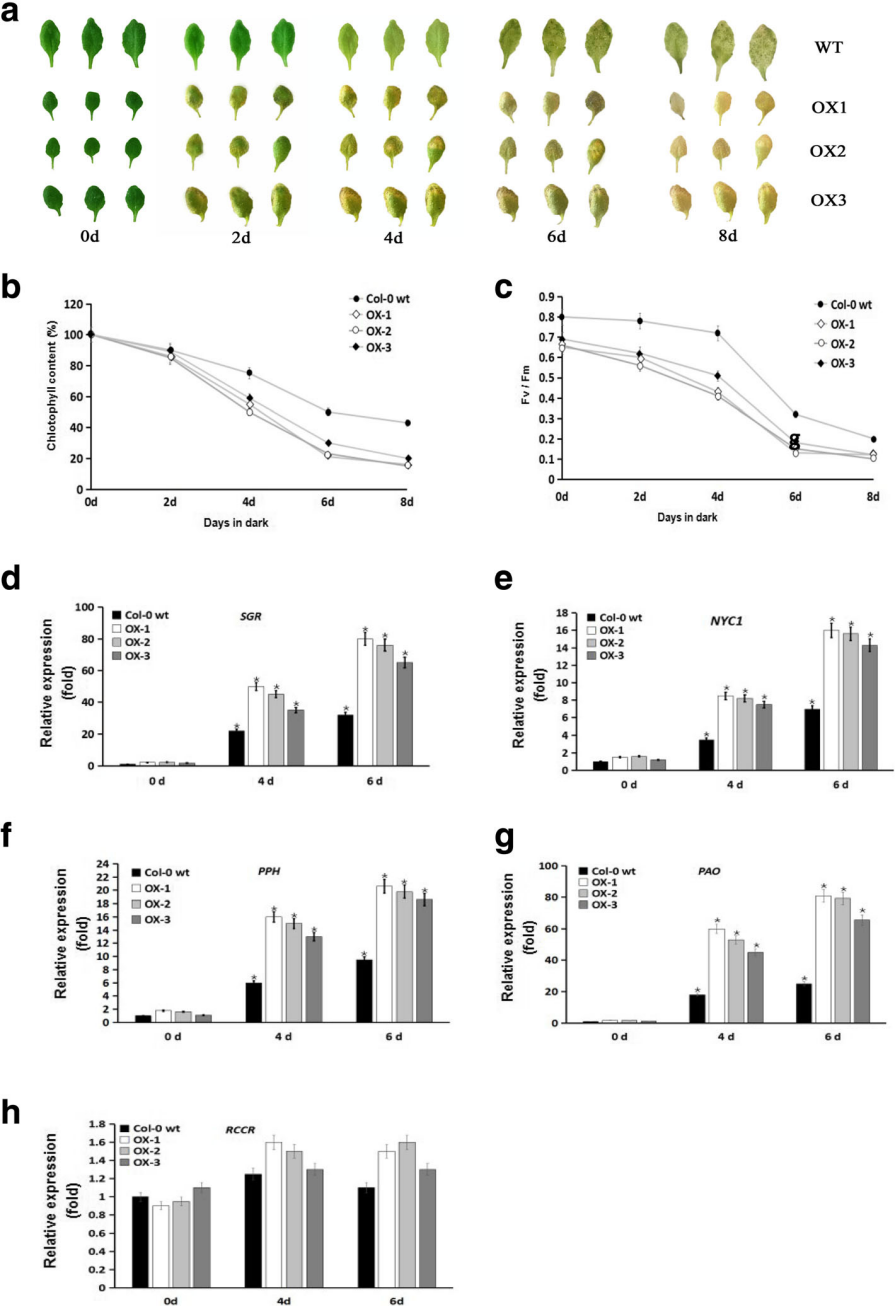
Analysis of phenotypes, chlorophyll contents, PSII photochemical efficiency (Fv/Fm), and expression levels of chlorophyll catabolism genes in detached leaves from WT and OX plants during dark-induced leaf senescence; **a** senescing phenotypes of detached leaves after 0, 2, 4, and 8 days in the dark; **b** chlorophyll contents in detached leaves from WT and OX plants were measured after 8-days dark treatment. **c** leaves were detached from WT and OX plants and Fv/Fm values were determined after 8-days dark treatment. **d**-**h** *SGR, NYC1, PPH, PAO*, and *RCCR* expression levels in detached leaves from WT and OX plants after 6-days dark treatment; *AtTublin* was used as an internal control. Asterisks (*) indicate significant differences; t-tests, *p* < 0.05

Because *IbVPE1* expression was dramatically increased during sweetpotato leaf senescence, we determined whether *IbVPE1* promotes leaf senescence. Chl catabolism is a clearly visible indicator of leaf senescence and fruit ripening, and the genes involved in Chl breakdown have been characterized in detail [24–27]. Thus, in the present study, we induced leaf senescence by placing detached leaves in the dark for 8 days and then investigated the roles of *IbVPE1* in senescence induced Chl degradation. Under these conditions, WT leaves remained greener than OX leaves, indicating greater Chl retention over 8 days in darkness (Fig. 6a). To confirm these observations quantitatively, we analyzed Chl and observed significantly lower Chl contents in OX leaves than in WT leaves after only 2 days in the dark (Fig. 6b). Thus, to investigate whether decreases in chlorophyll contents were accompanied by changes in photosynthetic activities, we determined maximal quantum yields of photosystem II (PSII). In these experiments, WT leaves had typical Fv/Fm values of about 0.8, whereas the Fv/Fm ratios in OX leaves were around 0.65, suggesting relatively low PSII activity (Fig. 6c). In subsequent dark treatments, the Fv/Fm values in OX plants were decreased by an average of 40% compared with WT leaves after 4 days. However, Fv/Fm values were decreased by 15% in WT leaves after 4-days dark treatment and decreased further after 6 days, reflecting progressive impairment of PSII quantum efficiency (Fig. 6c). Previous studies show that the genes *SGR*, *NYC1*, *PPH*, *PAO*, and *RCCH*, are central to Chl breakdown. Accordingly, we showed that *SGR*, *NYC1*, *PPH*, and *PAO* expression levels were dramatically upregulated in OX leaves compared with WT leaves after dark treatments for 4 and 6 days (Fig. 6d-h). These results indicate that *IbVPE1* actively stimulates the Chl breakdown pathway and accelerates leaf senescence.

## Discussion

The VPE family of proteins was initially identified as a group of cysteine proteases with roles in the maturation of seed storage proteins in vacuoles [7, 28]. However, identification of structural and functional similarities with the apoptotic protein caspase-1 in tobacco plants led to consideration of the VPE family as mediators of plant cell death [12]. Subsequently, the roles of VPE in PCD were rapidly defined in experiments showing responses to various stress stimuli [29–32]. These studies obscured additional metabolic roles of VPEs, and these remain largely unknown. However, recent studies have demonstrated developmental roles of VPEs in tomato plants. In particular, suppression of *SlVPE5* using a specific RNAi decreased the activity of acid invertase, which is involved in the hydrolysis of sucrose. Moreover, they found that the activity of this hydrolase was not fully correlated with mRNA expression in their RNAi-*SlVPE5* plants [33], indicating post-transcriptional regulation of sucrose accumulation in fruit by *SIVPE5*. Similarly, post-transcriptional functions of *SlVPE3* were related to the regulation of fruit ripening and disease resistance in tomatoes. In this study, *SlVPE3* was required for cleavage of the serine protease inhibitor *KTI4*, which contributes to resistance against the fungal pathogen *B. cinereal* [34]. Hence, VPEs may harbor undiscovered physiological functions in plant development. Herein, we investigated the function of the sweetpotato VPE gene *IbVPE1* using a convenient Arabidopsis overexpression model, and characterized the roles of *IbVPE1* in plant development and leaf senescence. Although VPE has been associated with PCD during plant responses to environmental stress, pathogen infection, and sucrose accumulation [23, 33], our results demonstrate that transgenic expression of a sweet potato VPE (in *IbVPE1*) in *Arabidopsis* affects leaf morphology, flowering time and dark induced chlorophyll breakdown.

To identify functions of *IbVPE1*, we examined tissue and developmental stage specific expression patterns in sweetpotatoes. *IbVPE1* expression was observed in all tissues but varied dramatically with developmental stages, with significantly higher expression in developing leaves and roots, and the highest expression in senescent leaves. Thus, to confirm the roles of *IbVPE1* in development and senescence, we analyzed expression patterns of transgenic *IbVPE1* in Arabidopsis tissues at various developmental stages. These experiments showed *IbVPE1* expression in all tissues, and maximal expression in developing and senescent leaves. Relative *IbVPE1* expression levels in Arabidopsis roots differed from that in sweetpotato.

### *IbVPE1* affects leaf development and flowering time by regulating class I TCPs and *AP1*

Consistent with the roles of *IbVPE1* in leaf development, the present transgenic Arabidopsis plants had reduced rosette leaf sizes and numbers. Leaf sizes and shapes reflect cell division, differentiation, and expansion during leaf development [35, 36], and TCPs are well-characterized regulators of leaf development and determinants of leaf morphology [35, 37]. TCP genes encode a conserved domain with an amino acid non-canonical basic helix-loop-helix (bHLH), which is involved in DNA binding and dimerization [38, 39]. In Arabidopsis, 24 TCPs have been identified, and these were previously subclassified into class I (13 proteins) and class II (11 proteins) transcription factors based on sequence homology [39]. Among class I TCP genes, *AtTCP7, AtTCP8, AtTCP14, AtTCP15, AtTCP20, AtTCP21, AtTCP22*, and *AtTCP23* regulate the expression of genes that are involved in shoot apical meristem and leaf development, and have similar functions to class II TCP transcription factors [40]. In loss-of-function studies, these genes were associated with leaf size, morphology, and flowering time, and *TCP14* and *TCP15* were shown to modulate cell proliferation in developing leaf blades and in specific floral tissues [41]. In addition, knockout of the class I *TCP* gene *tcp23–1* reduced rosette leaf numbers and produced an early flowering phenotype [42] similar to that shown in the present study. Moreover, simultaneous mutation of *tcp8*, *tcp15*, *tcp21*, *tcp22*, and *tcp23* altered leaf development traits in a study of Arabidopsis plants [43], suggesting that the present morphological observations in *IbVPE1* OX plants reflect regulation of TCP transcription factors. Accordingly, the present transcriptional comparisons of 8 class I TCPs in leaves (Fig. 5a) showed dramatic changes in expression levels of the class II TCPs *TCP14*, *TCP15*, and *TCP23* in *IbVPE1* OX plants. The genes *TCP14*, *TCP15*, and *TCP23* were previously subclassified with *TCP8* and *TCP22* and were shown to be clustered on chromosome1 [41], likely leading to general function redundancies, as indicated by comparisons of single and multiple TCP mutants, which showed only subtle and context dependent effects of individual TCP factors on cell proliferation. Hence, phenotypes reflect cumulative effects of multiple TCPs on cell division, requiring coordination of regulatory signals. In addition, the present observations of reduced rosette leaf sizes and numbers in *IbVPE1* OX plants may follow a balance of decreased function and increased cell proliferation.

The present *IbVPE1* OX Arabidopsis plants also exhibited an early flowering phenotype, as shown in a previous loss-of-function study of *TCP23* [42]. Floral initiation is an important physiological switch from vegetative to reproductive states [44]. Subsequently, flowering times are strictly controlled by environment cues (day lengths, temperature, water, and nutrient availability) and endogenous signals (stages of development) to maximize reproductive success. Hence, whereas *IbVPE1* influenced endogenous signaling pathways that regulate flowering under the present normal long-day conditions, further studies are required to decipher direct and indirect effects, or identify the affected transcription factors and hormones. Herein, we determined the expression levels of 13 flowering-related genes and observed a 10-fold increase in the transcription of *AP1* and significant induction of *LFY* in *IbVPE1* OX plants. *AP1* reportedly activates the genes *LFY* and *CAULIFLOWER* to promote the formation of floral meristems (FM) [45–49]. In agreement, flower formation in the axils of sepals was inhibited in *AP1* mutants, resulting in failure to establish FMs [48, 50, 51]. Taken with our results, these studies suggest that *IbVPE1* alters the flowering times of Arabidopsis plants by up-regulating *AP1* and *LFY*. But, it needs to be uncoverd that whether *IbVPE* affects those transcript levels ditrectly or indirectly.

### Expression of *IbVPE* in Arabidopsis leads to accelerated chlorophyll breakdown via activation of chlorophyll catabolism during dark induced senescence

Numerous studies associate specific hormones and genes with initiation, promotion, and inhibition of senescence [52–57], and the induction of senescence likely reflects the combined effects of multiple factors. Among hormones of senescence, ethylene and cytokinin are known promoting and delaying factors, respectively, in higher plants [58, 59]. It is reported that several cysteine protease including vacuolar processing enzyme are highly induced by ABA-treatment in senescencing flower of daffodil [60] As is a strongly visible feature of leaf senescence, Chl breakdown is accompanied by degeneration of chloroplasts and disruption in photosynthesis [5, 6, 55, 56]. Although ethylene reportedly induces the expression of *SlVPE3*, which conversely regulates ethylene synthesis in tomatoes via a positive feedback loop [34], the functions of VPE in Chl catabolism and photosynthesis remain unknown.

Herein, we assessed dark-induced leaf senescence in *IbVPE1* OX Arabidopsis plants because this process appears continuously in single rosette leaves rather than in whole plants [61]. These experiments showed that expression of *IbVPE1* resulted in increased in transcription of genes encoding enzymes that are related to Chl catabolism, except for red Chl catabolite reductase (RCCR), during dark-induced leaf senescence. Similarly, Chl genes were reportedly upregulated in NO-deficient mutant nos1/noa1 plants with only slight reductions in *RCCR* expression during senescence, and RCCR activity was constant throughout leaf development [52, 62]. In agreement, the present chlorophyll contents and photosynthetic Chl fluorescence parameters (Fv/Fm) were decreased by *IbVPE1* OX in detached leaves compared with those in detached WT leaves.

## Conclusion

The present experiments demonstrate that *IbVPE1* influences flowering times, leaf development, and senescence as well as the expression of flowering-related genes, such as TCP transcription factors and genes encoding Chl catabolism enzymes. These data suggest that, in addition to roles in PCD, VPEs influence development and senescence in *Arabidopsis* leaves. Further studies are required to elucidate post transcription and protein–protein interactions of *IbVPE1* with developmental molecules in plants.

## Methods

### Plant materials and growth conditions

Branches of sweetpotato plants (*Ipomoea batatas* (L.) Lam. cv Xu18) with 6–8 fully developed leaves were collected from the Institute of Agricultural Science, Xuzhou, Jiangsu, and were used in analyses of *IbVPE1* expression levels. Leaves were divided into L1–L5 developmental stages according to sizes. L1–L5 stages are defined as unopened immature leaves, not fully-expanded immature leaves, fully-expanded mature leaves, and partial (early) and completely (late) yellowing senescent leaves, respectively. L3 mature leaves were collected from the position between 3 and 6 and were counted downward from the shoot apex. Various root samples, including fibrous roots (< 5 mm in diameter), pencil roots (< 15 mm in diameter), swollen roots (> 15 mm in diameter), and mature root, were collected at 4, 8, and 12 weeks, respectively, after planting.

Wild-type (Columbia, Col-0) and *IbVPE1* overexpressing plants (OX lines) were grown under long day 16-h light/8-h dark conditions at 23 °C under 60% humidity in a soil mixture. For tissue-specific expression analyses, roots, stems, and leaves were collected at 30 days, and flowers and silique were collected upon appearance. For analysis developmental stages of roots and leaves, specimens were collected at 10, 20, 30, 40, and 50 days after planting.

### Sequence and phylogenetic analyses

The *IbVPE1* sequence was obtained by screening and sequencing the Xu18 fosmid library constructed by the Institute of Integrative Plant Biology, School of Life Science, Jiangsu Normal University, Xuzhou. The amino acid sequence of *IbVPE1* was decoded using ExPaSy translation tools (http://web.expasy.org/translate/). Other published VPE protein sequences were obtained from several databases using BLAST searches with the *IbVPE1* gene sequence. A phylogenetic tree was constructed using the neighbor-joining method with MEGA 7.0 software. Amino acid sequences used to construct the tree are listed in Additional file 1: Figure S1.

### Construction of transgenes and plant transformation

Total RNA was isolated from sweet potato (Xu18) leaf sample using the Plant RNeasy Kit (Qiagen). cDNA was synthesized using GoScript™ Reverse transcription System (Promega). To generate *IbVPE1* overexpression plants using Col-0 as background, the full-length coding sequence (CDS) of *IbVPE1* was first amplified using the primers listed in Additional file 2: Table S1, and was then cloned into the cloning vector PBI121 via XbaI and SmaI restriction enzyme sites, and an *IbVPE* promotert was also cloned into PBI121:GUS via XbaI and SacI to generate PBI121-*proIbVPE:*GUS. *Arabidopsis* transformation was performed using vacuum infiltration [63] with the *Agrobacterium tumefaciens* GV3101 strain.

### Subcellular localization analysis

Subcellular localization analyse was performed with cloning the full length of the *IbVPE1* CDS into pCAMBIA1300-GFP vector and the protein was fused in-frame with GFP for expression under the control of the *CaMV35S* promoter. The fused construct PCMBIA1301-35S:*IbVPE1*-GFP were transformed into onion epidermis cell by infiltration with the *Agrobacterium tumefaciens* GV3101 strain and green fluorescent protein (GFP) signals were observed using a fluorescence microscope with 488-nm.

### Phenotypic analysis

Numbers of rosette leaves were counted at the stage of bolting after elongating 0.5 cm of the main stem. Although cotyledons were not counted, petiole lengths, blade lengths, blade widths, blade perimeters, and blade areas of the fifth leaves were determined at bolting stage. Data were analyzed using LeafJ [64] with ImageJ software (https://imagej.nih.gov/ij/) as described previously [24].

### Gene expression analysis

Total RNA was extracted from samples using a plant RNeasy extraction kit (Qiagen, Valencia, CA, USA). Transcription analyses of genes were conducted using real-time PCR with a Rotor-Gene Q real-time thermal cycling system (Qiagen, CA, USA), QuantiTect SYBR Green RT-PCR kits (Qiagen, CA, USA), 200 ng RNA samples, 35 cycles and the primers listed in Additional file 2: Table S1 as described previously [65, 66].

### Senescence induction

Dark-induced leaf senescence was achieved in leaves that were detached from plants for 3-weeks. The leaves were initially placed on petri dishes containing two layers of filter papers soaked in 15 ml of distilled water. Subsequently, petri dishes were covered with aluminum foil and were kept in the dark at 23 °C as described previously [52].

### Chlorophyll measurements

After dark-induction, chlorophyll was extracted from leaves using 80% (V/V) acetone and chlorophyll contents were then determined using a spectrophotometer at wavelengths of 645 and 663 nm as described previously [52, 67].

### Chlorophyll fluorescence measurements

The maximum photochemical efficiency of PSII was determined from the ratio of variable (Fv) to maximum (Fm) fluorescence (Fv/Fm) using a LI-6400XT Portable Photosynthesis system (LI-COR Biosciences, Lincoln, Nebraska USA) as described previously [52].

### Statistical analysis

All experiments were repeated three times and data are expressed as means ± SE. Differences between transgenic and wild-type (WT) plants were identified using Student’s t-test with SPSS Software (version 19.0), and were considered significant when *p* < 0.05.

## Highlights

*IbVPE1* regulates flowering times, leaf sizes and numbers, and senescence in *A. thaliana* plants.

Chlorophyll contents in *IbVPE1* OX plants decreased more quickly than in WT plants under the condition of dark-induced senescence.

## Additional files

Additional file 1: **Figure S1.** Amino acid sequences of the plant VPEs used to construct the phylogenetic tree and databases used to retrieve the VPE amino acid sequences. (PDF 280 kb)

Additional file 2: **Table S1.** Gene-specific primers used in full-length cDNA and RT-PCR analysis (PDF 80 kb)

## Abbreviations

AP1: APETALA1
bHLH: Basic helix-loop-helix
BN1/2: BRITTLE NODE1/2
CO: CONSTANS
FLC: FLOWERING LOCUS C
FM: Floral Meristems
FRI: FRIGIDA
FT: FLOWERING LOCUS T
GFP: Green Fluorescent Protein
LD: LU MINID EPENDENS
LFY: LEAFY
NYC1: NON-YELLOW COLORING1
PAO: PHEIDE A OXYGENASE
PCD: Programmed Cell Death
PPH: PHEOPHYTINASE
PSII: Photosystem II
RCCH: Red Chl catabolite reductase
SGR: STAY-GREEN
SOC1: Super of overexpression COI
TCP: Teosinite branched1/Cycloidea/Pcf
TMV: Tobacco Mosaic Virus
VPEs: Vacuolar Processing Enzymes
