# Supplementary material for: Expression of IbVPE1 from sweet potato in Arabidopsis affects leaf development, flowering time and chlorophyll catabolism
Source: BMC Plant Biol. 2019 May 6;19:184. doi: 10.1186/s12870-019-1789-8 (PMC6503384; doi:10.1186/s12870-019-1789-8)
Supplement: Supplementary file 1 — Figure S1. Amino acid sequences of the plant VPEs used to construct the phylogenetic tree and databases used to retrieve the VPE amino acid sequences. (PDF 280 kb) [file 12870_2019_1789_MOESM1_ESM.pdf]

Figure. S1. Amino acid sequences of the plant VPEs used to construct the phylogenetic tree and databases used to retrieve the VPE amino acid sequences. Short VPE names indicated on the detailed tree of Supplemental Figure S3 are shown. Sequences labeled with NCBI accession number, eg., >XP_XXXX, >NP_XXXX were retrieved from NCBI. Other sequences, eg., >MA_XXXX (Picea abies), >PITA_VVVVV (Picea taeda) were retrieved at http://congenie.org; >KDXXXX(Aegilops tauschii) , >AETXXXX (Triticum monococcum), >CBMXXXX(Papaver- rhoeaswas), >AESXXXX (Medicago truncatula), >ACGXXXX(Zea mays), >VvXXXX were retrieved from Genebank; Arabidopsis thaliana sequences were retrieved at http://www.arabidopsis.org/; Cucumis sativus sequences were retrieved at http://cucumber.genomics.org.cn/page/cucumber/index.jsp; Capsicum annuum sequences were retrieved at http://peppersequence.genomics.cn/page/species/index.jsp https://solgenomics.net/tools/blast?db_id=279; >AchnXXXX (Actinidia chinensis Planch) was retrieved at http://bioinfo.bti.cornell.edu/cgi-bin/kiwi/blast.cgi; >MDPXXXX(Malusdomestica) was retrieved at http://www.applegene.org/; >AGCXXXX (Malus sikkimensis) was retrieved at http://www.applegene.org/; >OsXXXX(Oryza sativa) was retrieved at http://www.ricedata.cn/gene/; >Solanum lycopersicum sequences were retrieved at https://solgenomics.net/tools/blast?db_id=279 ; >Solanum melongena, >Solanum tuberosum, >Petunia axillaris sequences were retrieved at https://solgenomics.net/tools/blast?db_id=279.

VPE* amino acid sequences*:

>XP_001759301.1 VPE Physcomitrella patens
MALSVEGIFLILLCSLGAMAVAAREWDGKIVMPTEEGSKDPQPTEDGQRWAVLVAGSSGY
GNYRHQADVCHAYQILKKGGMKDENIVVFMFDDIAHNRHNPRPGVILNHPNGEDVYHG
VPKDYTGKNVTVNNLLAVLLGDKKTLKGGSGKVVNSGPNDHIFIYYSDHGGPGVLGMPT
NPNLYADDLLKTFKKMHEAKTYKEMVFVVYIEACESGSIFQGLLPKDLNIYATTAANAEES
SWGTYCPGMFPAPLEEFDTCLGDLYSVAWMEDTEVENLKKETLRDQYMIVKSRTSNHNT
YKSGSHVLEFGDLKMKPEELDQYLGYDPANENVTGPIFLREYLAIRLGGVEERHINQRDA
DLVHYWHRYHKSKVGSTAKAEAELDLMRILSHRMYIDKSVDLVGRLLFGVEAGPTTLSA
VRPDGLPLTDDWACLKSMVSAFELSCGELSEYGMKHMRAFANICNAGVEPSKMSGVAAE
ACAVSAFGSGTLQIPTTGFSA

>XP_001769381.1 VPE Physcomitrella patens
MPTGEGHKGEEGTRWAILIAGSAGYWNYRHQADVCHAYQILKRGGLKDENIIVFMHDDI
AYHPENPYPGTIINKPDGPDVYQGVPKDYTGSDVTVSNLYAAILGDKSAIEGGTGKVVDS
GPNDHIFIYYSDHGGPGVLGMPNPPNLYADDFVGILKKKAAAGTFKELVIYLEACESGSIFE
GLLPEGLNIYVTTASNAVESSWGTYCPGMYPSPPSEYGTCLGDLYSVAWMEDTEKENLKK
ETLEDQYLIVKSRTSNHNTYRSGSHVMQYGDLKIDVEELERYLGFDPANENVTKPGLSEL
SPVNSDIVTHVPQREADLVHLKHKFYNAKKGSLREANAASELAKTILHRRHLDDSVRLIG
ELLFAGEDALQKLGAVRPAGSVVVDDWACLKNMVRIFEASCGPLTQYGMKHMRAFANIC
NAGINSSRMSLASLEVCKISTSVDLGIWSPVTSGFSA

>XP_001755207.1 VPE Physcomitrella patens
MPTEEDAEKGTRWAILIAGSSGYWNYRHQADVCHAYQILKRGGLKEENIVVFMYDDIAY

STENPHPGKIINKPDGPDVYQGVPKDYTGADVTVSNFYAALLGDKDAIKGGSGKVVNSG
PNDHIFIYYTDHGGAGVLGMPTSPNLYADDFVDTLKKKAAAGTFKELVIYLEACESGSIFE
GLLPEGLNIYVTTASNAEESSWGTYCPGMYPPPPPEYDTCLGDLYSVAWMEDTEIENLKK
ETLEDQYVIVKSRTSNHNTYRTGSHVMQYGDVKLDVEELARYLGYDPANENVTKPELPE
FLSAHTEILTHVDQREADLIHLRYKFRNAVKGSLREANAATELAKTIVHRKHLDDSVQLIG
EILFAGENALEKLTAVRPAGSVVVDDWACLKTMVRTFEASCGPLTQYGMKHMRAFANICN
ARIDPAKMAVASSEACKLSTSAGSGIWSPVTSGFSA

> XP_001784456.1 VPE Physcomitrella patens
MPIDFNSELKGVRWAILIAGSSGYGNYRHQADICHAYQILKRGGLKEENIVVFMYDDIAN
NEENPHRGKVFNKPYGPDVYPGVPKDYTGENITVSNFYAAILGDADATKGGSGKVVASGP
NDHVFIYYADHGGAGVLGMPNDPILYADEFVDTLKKKAAAGTFKKMVIYVEACESGSIFD
GLLPTGLNIYVTTASDPDENSWGTYCPTMIPPPPPEFGTCLGDLYSVSWMEDAEMENLKK
ETLNDQYRIVKSRTSDNDTYMTGSHVMQYGDIEIDAEEVERYLGFDPANENVTRPELPVS
KAPATASGMHVMQREAELLHLWHKYHKAVDGSKKESAGMELTRTIAHRMHVDNSIKLIG
DHMFGLDTSLLRLKAVRPAGQVLVDDWSCLKAMVRTFEASCGPLTQYGMKHMRAFASIC
NAGIDLDTMKKATSQACGFSETDTDLRTADSPRFSASAKEFVKTIEF

>MA_60392g0010 VPE Picea abies
MGFVINIVLYCVLFLGLNGVLLYARPDDEFAGNNVIRMPIYKQERHPSESKEEDEGGSISTR
WAVLLAGSSGYGNYRHQADVCHAYQILRKGGLKEENIIVFMYDDIANNSDNPRPGTIINH
PNGKDVYAGVPKDYTGDNVNVNNFLAVLLGDKSSVKGGSGKVVDSGPNDHIFIYYSDHG
GPGVLGMPIPPYLYAHDLVEVLKKKHAAGAYKEMVIYIEACESGSIFEGLLPKGLNIYVTT
AANGEESSWGTYCPGMYPPPPPEYETCLGDLYSVAWMEDSEKHNLKTETIKQQYQLVKF
RTSDHNTYQAGSHVMQYGDIPISKEHLFLYIGSDPANANATFIYDNGFPEFPDEKDVMRAV
NQRDADLLYLWQKYKRSKEGSIEKLESQKHMVDLMTHRMHLDKSVNLIGKLLFGSVRG
LNVLNTVRPPGQPLVNDWDCLKTMVRTFEKHCGSLSQYGMKHMRSLANICNEGVTKNT
MAVVSAEACNQMSSRFRTSLHRGFSA

>MA_86205g0010 VPE Picea abies
MFSVSLFLLFCLLGFHVIVDASRFHFDSKIRLSTAGEDEDIGTQWAVLLAGSAGYSNYRHQ
ADVCHAYQILKRGGLKDENIVVFMYDDIANNPVNPRPGIIINHPEGSDVYAGVPKDYTGK
EVTVDNFFAVILGDKDSVKGGSGKVVDSGPNDHIFIYYTDHGGPGVLGMPSGHMLYAKD
LIDVLKKKHAADTYKQMVIYVEACESGSIFEGLLPEGMNIYVTTASNAEESSWGTYCPGM
KPSPPLEYDTCLGDLYSVAWMEDSEVHNTMKETLKQQYQVVKERTSNHQTYGMGSHVM
QYGDIPISEDPLSLYIGFDPANADAIFENRLPQYLREKDAAAINQRDADLLYLWQKYKRSK
PDSTEKLEAQQELIESMSHRLYLDKSINFIGKILFGSDTGTAVLNAVRPSGQPLVDDWDCLK
TMVRTFESHCGSLSQYGMKHVRALANICNNGVSVDTMAEVSAEACTQISSGKWRSLQV
GYSA

>PITA_000008627 VPE Picea taeda
MMRLCTSAMGSSKGFQLGLLCYFLLLLLDSSKVADGARRGWDSVLKMPTDHIDDDSDAI
GTQWAILLAGSSGYWNYRHQADICHAYQILRKGGLKEENIVVFMYDDIAQDEENPNPGTI
INHPQGSDVYAGVPKDYTGENVTVNNFFAVLLGNKSLVTGGSGKVVESDPNDRIFIYYSD

HGGPGVLGMPLPPYLYANDFIQVLKQKHAAGSYREMVIYIEACESGSIFEGLLPTDMNIYA
TTASNAEENSWGTYCPGMDPPPPPEYDTCLGDLYSVAWMEDSEMNNLKEETLLQQYNLV
KRRTSNHNTYMTGSHVMQYGNLTISQEELYLYIGFDSANSNASLVLENTLLLERTEAKAIN
QRDADLLYMWQKYKKSKEDSPERFTAQTQLLEFMAHRMHVDKSVKLVGNLLFGPEKGS
AVLKAVRPQGEPLVDDWDCLKKMVRTFERHCGSLAQYGMKHMRALANICNEGISMDTM
ATVSAEACTQFPAGSWSSLQRGYSA

>PITA_000069534 VPE Picea taeda
MGFINNMVIVLSCVLFLGLNGVLLHARPDEFTGDGFIRMPVDKQHSFESKEEEDGDSIGTR
WAVLLAGSSGYGNYRHQADVCHAYQILRKGGLKEENIIVFMYDDIANNSDNPRPGTIINH
PNGKDVYAGVPKDYTGDNVNVNNFLAVLLGDKSSVKGGSGKVVDSGPNDHIFIYYSDHG
GPGVLGMPIPPYLYAHDLVEVLKKKHAANAYKEMVIYIEACESGSIFEGLLPKGLKIYVTT
AANGEESSWGTYCPGMYPPPPPEYETCLGDLYSIAWMEDSEKHNLKTETIKQQYQLVKFR
TSDHNTYRAGSHVMQYGDIPISKEQLFLYIGSDPANANATFTYDNGFPEFPDEKDVRAVNQ
RDADLLYLWQKYKRSKEGSIKKLESQRQMVDLMTHRMHVDKSMNLIGKLLFGSARGPN
VLNTVRPPGQPLVDDWDCLKTMVRTFEKHCGSLSQYGMKHMRSLANMCNEGVTKNTM
TAVSAEACNQMPSRFRASLHRGFSA

>PITA_000008629 VPE Picea taeda
MGSVMFSLCLFLLFCLLGFHGIGIVDASRLHFDSKIRLSTDGKDENLGTQWAVLLAGSAG
YSNYRHQVIFCMVVPTKVCIMQADVCHAYQILKRGGLKDENIIVFMYDDIANNPANPRPG
IIINHPEGSDVYAGVPKDYTGKEVTVDNLFAVILGDKDSVKGGSGKVVDSGPNDHIFIYYT
DHGGPGVLGMPIGPSLYANDLIDVLKKKHAADSYKQMVIYVEACESGSIFEGLLPEGMNI
YVTTASNAEESSWGTYCPGMEPSPPLEYDTCLGDLYSVAWMEDSEVHNTMKETLDQQYQ
VVKERTSNHHTYGMGSHVMQYGDIPISEDPLSLYIGFDPANANVTFENSLPQYLREKDAT
VINQRDADLLYLWQKYKRSKPDSTEKLEAQQELIESMTHRLHLDKSINFIGKLLFGSEMGT
TVLKAVRPSGQPLVDDWDCLKTMVIFKCSMVICISSVLQPTRL

>KD558704.1 VPE Aegilops tauschii
MAMASFRPLPLALLLAACLSAFVLAVAHARTPGLDPTIRLPSQRAAGQEDDDSVGTRWAV
LIAGSNGYYNYRHQADICHAYQIMKKGGLKDENIIVFMYNDIAHNPENPRPGVIINHPQG
GDVYAGVPKDYTGKEVNVKNFFAVLLGNKTAVSGGSGKVVDSGPNDHIFVFYSDHGGPG
VLGMPTYPYLYGDDLVDVLKKKHAAGTYKSLVFYLEACESGSIFEGLLPNDIGVYATTAS
NAEESSWGTYCPGEYPSPPPEYDTCLGDLYSISWMEDRANLMAMELGASFAVNAPCRNS
CSCCTQPYSYEIATEHHLDVHNLRTESLKQQYNLVKKRTAAQDSYSYGSHVMQYGSLDL
NAEHLFSYIGSNPANENTTFVEDNALPSFSRAVNQRDADLVYFWQKYRKLAESSPEKNDA
RKQLLEMMGHRSHIDNSVELIGNLLFGSAGGPMVLKAVRPAGEPLVDDWSCLKSTVRTFE
SQCGSLAQYGMKHMRSFANICNAGIVPEAMAKVAAQACTSIPTNPWSATHKGFSA

>XP_003569218.1 VPE Brachypodium distachyon
MASFRLLPLALLLCACLSAHARTSLLEQTIRLPSQRGAAGQQEVDDDSVGTRWAVLIAGS
NGYYNYRHQADICHAYQIMKKGGLKDENIIVFMYDDIAHNPENPRPGVIINHPQGGDVYA
GVPKDYTGKEVNVKNFFAVLLGNKAAVSGGSGKVVDSGPNDHIFVFYSDHGGPGVLGMP
TYPYLYGDDLVDVLKKKHAAGTYKSLVFYLEACESGSIFEGLLPNDIGIYATTASNAEESS

WGTYCPGEYPSPPPEYDTCLGDLYSIAWMEDSDVHNLRTESLKQQYDLVKKRTAPENSYS
YGSHVMQYGSLDLNAEHLFLYIGSNPANDNTTFVEGNSLPSFSRAVNQRDADLVYFWQK
YRKLAESSPAKNDARKELLEMMAHRSHVDNSVELTGNLLFGSEDGPMVLKTVRTAGEPL
VDDWGCLKSTVRAFESQCGSLAQYGMKHMRSFANICNAGILPEATAKVAAQACPSIPANP
WSATHKGFSA

>XP_003569976.1 VPE Brachypodium distachyon
MAAWWCFALLLVLCAPAGADVSKGKWEPLIRMPGEKEPATARGFEGPEEEDGVGTRWAV
LIAGSSGYGNYRHQADICHAYQVLRKGGLKEENIVVFMYDDIANSALNPRPGVIINHPQG
EDVYAGVPKDYTGEQVTAKNLYAVLLGNKTAVTGGSKKVIDSQPKDHIFIYYSDHGGPGV
LGMPNLPYLYAGDFIKILQQKHASNTYAKMVIYVEACESGSIFEGLMPADLNIYVTTASNA
EESSWGTYCPGMEPSPPSEYITCLGDLYSVSWMEDSENHNLKEETIKKQYEVVKRRTSDL
NSYSAGSHVMEYGDKTFKDEKLYLYQGFNPANANITNKLFWQAPRAAINQRDADLLFLW
RRYEMLHEKSKEKVKVLREISETVMHRKHLDNSVDLIGQLLFGFENGPSVLQAVRPSGKP
LVDDWDCLKRMVRIFESHCGPLTQYGMKHMRAFANICNNGIPGSTMKEGSISACGSRNIA
RWSPLIQGYSA

>XP_006646004.1 VPE Oryza brachyantha
MAPAPRLLPLLPPLAALLLCAHLAVAVARPRMEPWGSRGPRMEPWDSSDNLLLPSEREWE
RERERSVEDEDAAEAAVGTRWAVLIAGSNGYYNYRHQADVCHAYQIMKKGGLKDENIIV
FMYDDIAHNSENPRPGVIINHPQGGDVYAGVPKDYTGKDVNVKNLFAVLLGDKTAVSGG
SGKVLDSGPNDHIFIFYSDHGGPGVLGMPTYPYLYGDDLVDVLKKKHAAGTYKSLVFYLE
ACESGSIFEGLLPDDINVYATTASNADESSWGTYCPGEYPSPPPEYDTCLGDLYSVAWMED
CDVHNLRTESLRQQYNLVKERTSVQHTYDSGSHVMQYGSIELNAHHLFLYMGSNPANDN
STFVEDNSLPSFSRAVNQRDADLVYFWQKYRKLAEGSPEKNEARKQLLEMMAHRSHVD
NSVELIGNLLFGSEEGPRVLKAVRATGEPLIDDWSCLKSMVRAFEAQCGSLAQYGMKHM
RSFANICIAGISAEEMAKVAAQACTSIPSNPWSSTHRGFSA

>AET08891.1 VPE4 Triticum monococcum
MAMASFRPLPLALLLAACLSALVLAVAHARSPRLEPTIRLPSQRAAGQEDDDSVGTRWAV
LIAGSNGYYNYRHQADICHAYQIMKKGGLKDENIIVFMYDDIAHNLENPGPGVIINHPQG
GDVYAGVPKDYTGKEVNVKNLFAVLLGNKTAVSGGSGKVVDSGPNDHIFVFYSDHGGPG
VLGMPTYPYLYGDDLVDVLKKKHAAGTYKSLVFYLEACESGSIFEGLLPNDIGVYATTAS
NAEESSWGTYCPGEYPSPPPEYDTCLGDLYSISWMEDSDVHNLRTESLKQQYNLVKKRTA
AQDSYSYGSHVMQYGSLDLNAEHLFSYIGSNPANENTTFVEDNALPSFSRAVNQRDADLV
YFWQKYRKLAESSPEKNDARKQLLEMMGHRSHIDNSVELIGNLLFGSAGGPMVLKAVRP
AGEPLVDDWSCLKSTVRTFESQCGSLAQYGMKHMRSFANICNAGIVPEATAKVAAQACTS
IPTNPWSATHKGFSA

> ACG34144.1 VPE Zea mays
MVAARLRLALLLSVCLCSAWARPRLETAIRLPSQRAAAADETDDGAVGTRWAVLIAGSSG
YYNYRHQADICHAYQIMKKGGLKDENIIVFMYDDIAHSPENPRPGVIINHPQGGDVYAGV
PKDYTGRDVNVDNFFAVLLGNKTALRGGSGKVVDSGPDDHIFVFYSDHGGPGVLGMPTY
PYLYGDDLVDVLKKKHAAGTYKSLVFYLEACESGSIFEGLLPNDINVYATTASNAEESSWG

TYCPGEFPSPPPEYDTCLGDLYSVAWMEDSDFHNLRTESLKQQYKLVKDRTAVHDTFSYG
SHVMQYGALELNVQRLFSYIGTDPANDGNTFIEDNSLPSFSKAVNQRDADLVYFWQKYR
KLADSSPPKSEARKELLEVMAHRSHVDSSVELIGSLLFGSEDGPRVLKAVRAPGEPLVDD
WSCLKSIVRTFEARCGSLAQYGMKHMRSFANMCNAGILPEAVSKVTAQACSSIPSNPWSSI
HKGFSA

> CBM41515.1 Papaver rhoeas
MVKFLFSVIILFFLLSAVGSSARNIEEDGVIRLPSEVKDFINGKNIDDDSVGGTRWAVLIAGS
SGYWNYRHQADVCHAYQVLKRGGVKDENIVVFMYDDIALNEENPRPGVIINHPKGEDVY
AGVPKDYTGRDVTAHNFYSVLLGNKTAVKGGSGKVIDSGPNDHIFIYYSDHGGPGVLGM
PTYPYLYADDLVNVLKQKHALGAYKSLVFYLEACESGSIFEGILPKGLNIYATTASNAEESS
WGTYCPGEFPSPPSEYETCLGDLYSVAWMEDSDVHNLRSETLKQQYHLVKERTQNANSA
YGSHVMQYGDLEVSKEDLFLYMGTNPANDNNKFIEQNSLPSLSGSVNQREADLIHFWQK
YRKAPEGSQRKADAQKQFVEVMAHRMHVDHSIKLIGKLLFGFEKGPQVLEAVRPAGQPL
VDDWDCLK

>AES80307.2 VPE Medicago truncatula
MNQIISCWGALITIVWMSVTVTLSKGVVRPMVHKHDEQGNFEVVGKKWALLVAGSKGY
SNYRHQSNICHAYHILKSGGLQDENIIVFMYDDIAYHNENPRPGVIINRPDGPNVYPGVPK
DYTGNNTNAENFFAVLNGNLSGITGGSGKVLNSDPNDTIFIYYSGHGYPGLIGMADQSLVY
AKDLVDALKKKHASNSYKKMVIYVEACYSASLFEGLLPNNISIYVTTSANARELGYGFYC
PGSINLSSTEYTTCLGDTFGISWMEDSDKNESTNETLQQQYVTVRDRTITSHVTQLGDLNI
SNDFLDTYIGSAPLNNVSDNYNLTNTTSVYSFEPFNTSTSLVNQDDAYLLHLKLKLEKAVD
GSKDKLKAQNELDAEIAHRKHVDHNIHLIGNILFGEKKSSIMMSDLRSAGQPLIDDWNCL
KILFKTYESHCGILLSTYGRKYSRVFAYMCNIGIFEKQTISAVSQVCSRIHHSS

>AES78483.1 VPE Medicago truncatula
MNHKNKYWVALIASIWMSVTDNVFAEGESTTGKKWAFLVAGSNGYVNYRHQADICHAY
QILKKGGLKDENIVVFMYDDIAYNPQNPRRGVLINHPNGSDVYNGVPKDYIGDYGNLENF
LAVLSGNKSATKGGSGKVLDTGPDDTIFIFYTDHGSPGSIGIPDGGLLYANDFVDALKKKH
DAKSYKKMVIYMEACEAGSMFEGLLPNDINIYVTTASNKSENSYGFYCPNSYLPPPPEYDI
CLGDLYSISWMEDSEKNDMTKEILKEQYETVRQRTLLSHVLQYGDLNISNDTLITYIGADP
TNVNDNFNVTSTTNVFSFDDFKSPNPTRNFGQRDAHLIYLKTKLGRASSGSEDKLKAQKE
LEVEIARRKHVDNNVHQISDLLFGEEKGSIVMVHVRASGQPLVDNWDCLKTLVKTYESH
CGTLSSYGRKYLRAFANMCNNGITVKQMVAASLQACLEKN

>XP_003589032.1 VPE Medicago truncatula
MDSSTITVITIAAVSGSRDGDYIRSSASRHNDDNDGTRWAIAGSNGYWNYRHADVCHAYR
KGGKNIIVMYDDIASNVNRGVIINKDGGDVYGVKDYTGAVHADNYAAGNKSATGGSGK
VVDSGNDHIVYYTDHGGGVGMVGYYASDNVKKKHASGSYKSVYKISISMRTASNAVSS
WGTYCGYYSTCGDYSIAWMDSDIHNRTSHYKVKDRTINGYYGSHVMYGDVGSNNHYGT
NANDNISVDSSKRSSTAVNRDADIHWDKRKAGSRKNAKVAMSHRMHVDNSVKIGKGIK
GTDNVRAGSVDNWDCKTMVKTTHCGSSYGMKHMRSANICNAGITMAASA

>XP_003603121.1 VPE Medicago truncatula
MARFLFLIIATLIPIFSAATATAGDDFLRLPSQASRFFQSDDDNNEGTKWAILIAGSNGYWN
YRHQSDVCHAYQVLRKGGLKEENIIVFMYDDIADNQENPRPGVIINSPHGDDVYKGVPKD
YTGDDVNVNNFFAALLGNKSALTGGSGKVVDSGPNDHIFIYYSDHGGPGVLGMPTGPFM
YATDLIEVLKKKHASETYKSLVFYLEACESGSIFEGLLPEGLNIYATTAANAEESSWGTYCP
GENPSPPPEYETCLGDLYSVAWMEDSDIHNLQTETLHQQYELVKERTSNGNSIYGSHVMQ
FGDIGLSRDSLFLYLGSNPANENFTFMGRNSLVPPSKTVNQRDADLIHFWDKFRKAPQGSP
RKVAAQKQVLEAMSHRMHIDESIKLVGKLLFGMKKGPEVLASVRPAGQPVVDDWDCLK
SLVRTFETYCGSLSQYGMKHMRSFANFCNAGIHSEQMAEASAQACINIPANPWSSLHGGF
SA

>AT2G25940.1 αVPE Arabidopsis thaliana
MTTVVSFLALFLFLVAAVSGDVIKLPSLASKFFRPTENDDDSTKWAVLVAGSSGYWNYRH
QADVCHAYQLLKKGGVKEENIVVFMYDDIAKNEENPRPGVIINSPNGEDVYNGVPKDYT
GDEVNVDNLLAVILGNKTALKGGSGKVVDSGPNDHIFIYYSDHGGPGVLGMPTSPNLYAN
DLNDVLKKKYASGTYKSLVFYLEACESGSIFEGLLPEGLNIYATTASNAEESSWGTYCPGE
DPSPPSEYETCLGDLYSVAWIEDSEKHNLQTETLHEQYELVKKRTAGSGKSYGSHVMEFG
DIGLSKEKLVLFMGTNPADENFTFVNENSIRPPSRVTNQRDADLVHFWHKYQKAPEGSAR
KVEAQKQVLEAMSHRLHVDNSILLIGILLFGLEGHAVLNKVRPSGEPLVDDWDCLKSLVR
AFERHCGSLSQYGIKHMRSIANMCNAGIQMRQMEEAAMQACPTIPTSPWS SLDRGFSA

>AT1G62710.1 βVPE Arabidopsis thaliana
MAKSCYFRPALLLLLLVLLVHAESRGRFEPKILMPTEEANPADQDEDGVGTRWAVLVAGSS
GYGNYRHQADVCHAYQILRKGGLKEENIVVLMYDDIANHPLNPRPGTLINHPDGDDVYA
GVPKDYTGSSVTAANFYAVLLGDQKAVKGGSGKVIASKPNDHIFVYYADHGGPGVLGMP
NTPHIYAADFIETLKKKHASGTYKEMVIYVEACESGSIFEGIMPKDLNIYVTTASNAQESSY
GTYCPGMNPSPPSEYITCLGDLYSVAWMEDSETHNLKKETIKQQYHTVKMRTSNYNTYSG
GSHVMEYGNNSIKSEKLYLYQGFDPATVNLPLNELPVKSKIGVVNQRDADLLFLWHMYRT
SEDGSRKKDDTLKELTETTRHRKHLDASVELIATILFGPTMNVLNLVREPGLPLVDDWECL
KSMVRVFEEHCGSLTQYGMKHMRAFANVCNNGVSKELMEEASTAACGGYSEARYTVHP
SILGYSA

>AT3G20210.1 δVPE Arabidopsis thaliana
MSSPLGHFQILVFLHALLIFSAESRKTQLLNDNDVESSDKSAKGTRWAVLVAGSNEYYNYR
HQADICHAYQILRKGGLKDENIIVFMYDDIAFSSENPRPGVIINKPDGEDVYKGVPKDYTK
EAVNVQNFYNVLLGNESGVTGGNGKVVKSGPNDNIFIYYADHGAPGLIAMPTGDEVMAK
DFNEVLEKMHKRKKYNKMVIYVEACESGSMFEGILKKNLNIYAVTAANSKESSWGVYCP
ESYPPPPSEIGTCLGDTFSISWLEDSDLHDMSKETLEQQYHVVKRRVGSDVPETSHVCRFG
TEKMLKDYLSSYIGRNPENDNFTFTESFSSPISNSGLVNPRDIPLLYLQRKIQKAPMGSLESK
EAQKKLLDEKNHRKQIDQSITDILRLSVKQTNVLNLLTSTRTTGQPLVDDWDCFKTLVNSF
KNHCGATVHYGLKYTGALANICNMGVDVKQTVSAI EQACSM

>AT4G32940.1 γVPE Arabidopsis thaliana

MATTMTRVSVGVVLFVLLVSLVAVSAARSGPDDVIKLPSQASRFFRPAENDDDSNSGTRW
AVLVAGSSGYWNYRHQADICHAYQLLRKGGLKEENIVVFMYDDIANNYENPRPGTIINSP
HGKDVYQGVPKDYTGDDVNVDNLFAVILGDKTAVKGGSGKVVDSGPNDHIFIFYSDHGG
PGVLGMPTSPYLYANDLNDVLKKKHALGTYKSLVFYLEACESGSIFEGLLPEGLNIYATTA
SNAEESSWGTYCPGEEPSPPPEYETCLGDLYSVAWMEDSGMHNLQTETLHQQYELVKRRT
APVGYSYGSHVMQYGDVGISKDNLDLYMGTNPANDNFTFADANSLKPPSRVTNQRDADL
VHFWEKYRKAPEGSARKTEAQKQVLEAMSHRLHIDNSVILVGKILFGISRGPEVLNKVRS
AGQPLVDDWNCLKNQVRAFERHCGSLSQYGIKHMRSFANICNAGIQMEQMEEAASQAC
TTLPTGPWSSLNRGFSA

>VV09G09760 VPE Vitis vinifera
MNYYIVGILLFITLFSPFTKSEPPKLDRPSNETEFIHGNSKPNCKAAEPAKGKQWAVLIAGS
TDYENYRHQADICHAYQILKKGGLKDENIIVFMYDDIAFNVENPRPGVIINQPGGDDVYE
GVPKDYTQSAATVANVFAVLLGNKTAVQGGSGKVLDSGPDDHVFIYYADHGATGIIGMTD
GLIYAKDLIDVLKKKHEAKAYKTMVIYIEACEAGSMFQGLLPNNWDIYATTAANAEENSY
GTYCPDDYPSAPSEYDTCLGDTYSVAWLEDSEMHDLRFETLEKQYKTIRRRVFTQDLDFN
SHVTQYGDMKLSKEFLFTYMGTNPDNDNYTSMANSKPSGFSSASQYDAELLHFWYKFH
RAPEGSTRKLEAQKELHRKISHRMHVDHSMKEIGKLILGSENSTMMLLKTVRPLDQPVV
DDWDCYKMLVKTYEEHCGSLSRYGLKYTRALANMCNAGIKMEQMAVASAQACAKIKP

>VV04G07860 VPE Vitis vinifera
MTIFPAAVAAFLALSTLVAGGRHFAGDNGLLLPSEASRFFRPGGAADDDTGAESAGTRWA
VLIAGSNGYWNYRHQADICHAYQLLKKGGLKDENIIVFMYDDISFNEENPRPGIIINSPHG
EDVYEGVPKDYTGEDVTVDNFFAVILGNKTALSGGSGKVLDSGPNDHIFIYYSDHGGPGV
LGMPTSPYLYANDLIEVLKKKHASGTYNSLVFYLEACESGSIFEGLLPEGLNIYATTAANAE
ESSWGTYCPGEDPSPPPEYETCLGDLYSVAWMEDSDVHNLRTETLRQQYELVKKRTANDN
SVYGSHVMQYGDLGLNKEDLVLYMGTNPANDNYTFVDNNSLRLPSKAVNQRDADLVHF
WDKFRKAPEGSPRKAEAQKQFLEAMSHRTHIDHAIKLVGRLLFGMKKGSEVLKTVRPAG
QPLVDDWHCLKTLVRTFEAHCGSLSQYGMKHMRSIANICNAGIEKEQMAEASAQACVTIP
PGPWSSLDKGFSA

>XP_004501477.1 VPE Cicer arietinum
MYRFPTPTLLFLIVTLIALVSSNPEDFLRLPSESSRFFHSPSADDKENNEGTRWAILIAGSNG
YWNYRHQSDVCHAYQVLRKGGLKEENIVVFMYDDIAFNEENPRPGVIINSPHGDDVYKG
VPKDYTGEDVNVDNFFAALLGNKSALTGGSGKVVDSGPNDHIFIYYSDHGGPGVLGMPT
SPYMYASDLIEVLKKKHASGTYKSLVFYLEACESGSIFEGLLPEGLNIYATTAANADESSW
GTYCPGEFPSPPPEYETCLGDLYSVAWMEDSDMHNLQSETLHQQYELVKERTKNGNTLYG
SHVMQYGDIGLSENSLFLYLGTNPANENFTFVGRNSLVPPSKAVNQRDADLVHFWDKFRK
APQGSPRKAAAEKQVLEAMSHRMHIDDSIKLVGKLLFGMEKGPEVLTSVRPAGQPLADD
WNCLKTLVRTFETYCGSLSQYGMKHMRSFANFCNAGIHKEQMAEASAQACVNVPANPW
SSLRSGFSA

>NP_001236564.1 VPE2 Glycine max

MPTFFLPTLLLLLIAFATSVSGRRDLVGDFLRLPSETDNDDNFKGTRWAVLLAGSNGYWN
YRHQADVCHAYQILRKGGLKEENIIVFMYDDIAFNGENPRPGVIINKPDGGDVYKGVPKD
YTGEDVTVDNFFAALLGNKSALTGGSGKVVDSGPDDHIFVYYTDHGGPGVLGMPAGPYL
YADDLIEVLKKKHASGTYKNLVFYLEACESGSIFEGLLPEDINIYATTASNAEESSWGTYCP
GEYPSPPPEYTTCLGDLYSVAWMEDSDRHNLRTETLHQQYKLVKERTISGDSYYGSHVMQ
YGDVGLSRDVLFHYLGTDPANDNFTFVDENSLWSPSKPVNQRDADLIHFWDKFRKAPEG
SLRKNTAQKQVLEAMSHRMHVDNSVKLIGKLLFGIEKGPEVLNAVRPAGSALVDDWHCL
KTMVRTFETHCGSLSQYGMKHMRSFANICNVGIKNEQMAEASAQACVSIPSNPWSSLQR
GFSA

>XP_003550283.1 VPE Glycine max
MATLLLPTLLLLIPFATLVSARPHLAGDFLRLPSETDNDDNVQGTRWAVLLAGSNGYWNY
RHQADVCHAYQILRKGGLKEENIIVFMYDDIAFNGENPRPGVIINKPDGGDVYEGVPKDY
TGEDVTVGNFFAALLGNKSALTGGSGKVVDSGPDDHIFVYYTDHGGPGVLGMPAGPYLY
ADDLIEVLKKKHASGTYKNLVFYLEACESGSIFEGLLPEDINIYATTASNAEESSWGTYCPG
EYPSPPPEYSTCLGDLYSVAWMEDSDRHNLRTETLHQQYKLVKERTISGDSYYGSHVMQY
GDVRLSSDVLFHYLGTDPANDNFTFVDENSLWSPSKPVNQRDADLIHFWDKFRKAPEGSL
RKNAAQKQVLEAMSHRMHVDNSVKLIGKLLFGIEKGPEVLNAVRPAGSALVDDWHCLK
TMVRTFETHCGSLSQYGMKHMRSFANICNVGIKNEQMAEASAQACVSIPSNPWSSLQRGF
SA

>AGC94757.1 VPE Malus sikkimensis
MTRLASAVVLLFFASVLASAAGSRDLIGDILRLPSEASKFFGRGDDAPDQQDDGTVGTRW
AVLIAGSNGYWNYRHQADICHAYQLLKKGGLKDENIVVFMYDDIAYNEENPRQGVIINSP
HGSDVYEGVPKDYTGEDVTVNNFFAAILGNKTALTGGSGKVVDSGPNDHIFIYYTDHGGP
GILGMPTSPYIYANDLIEVLKKKHAAGTYKSLVFYLEACESGSIFEGLLPEGLNIFATTASNA
EESSWGTYCPGEYPSPPPEYDTCLGDLYSVAWMEDSDVHNLRSETLHQQYELVKTRTAND
NSGFGSHVMQYGDVGLSKNNLFVYMGTNPANDNHTFLGENSLRPSSKAVNQRDADLLH
FWHKYRKAPEGSARKIQAQKDFVEAMSHRMHIDQTMKLIGKLLFGIEKGPQVLNAVRPA
GQPLVDDWDCLKTMVRSFETHCGSLSQYGMKHMRSLANICNAGMTQDQMAEASAQAC
VSAPSGRWSSLHRGFSA

>XP_002516472.1 VPE Ricinus communis
MTIRLSTGIILILLTLCGVVSSSRDIVGDVIRLPSEASRFFRPADGKNGDDDSAGTRWAILIA
GSNGYWNYRHQADVCHAYQLLRKGGLKEENIIVFMYDDIAYNEENPRQGIIINNPHGEDV
YKGVPKDYTGENVTVGNFFAAILGNRTALTGGRGKVVDSGPNDHIFVYYTDHGGPGVLG
MPTNPYLYANDLIDVLKKKHASGTYKSLVFYLEACESGSIFEGLLPEGLNIYATTASNAEES
SWGTYCPGEYPSPPPEYETCLGDLYSIAWMEDSDVHNLQTETLHQQYELVKRRTSNGNSA
YGSHVMQYGDVGLSRENLFLYMGTNPANDNYTFVDENSLTPPSKAVNQRDADLVHFWD
KYRKAPDGSARKDQAQKQFVEAMSHRMHIDHSVKLIGKLLFGLEKASEVLSTVRPAGQP
LVDDWDCLKKLVRTFETHCGSISQYGMKHMRSLANLCNAGIREEQMAEASAQACITFPS
GPWSSLHKGFSA

>NP_001310660.1 VPE Ricinus communis

METHKSLLFFTNYVLFLVFTLSFLPIPGLLASRLNPFEPGILMPTEEAEPVQVDDDDQLGTR
WAVLVAGSMGFGNYRHQADVCHAYQLLRKGGLKEENIIVFMYDDIAKNELNPRPGVIINH
PQGEDVYAGVPKDYTGEHVTAKNLYAVLLGDKSAVQGGSGKVVDSKPNDRIFLYYSDHG
GPGVLGMPNLPYLYAMDFIEVLKKKHAAGGYKKMVIYVEACESGSIFEGIMPKDVDIYVT
TASNAQESSWGTYCPGMEPSPPPEFTTCLGDLYSVAWMEDSESHNLKKETVKQQYSSVK
ARTSNYNTYAAGSHVMQYGNQSIKADKLYLFQGFDPASVNFPPNNAHLNAPMEVVNQR
DAELHFMWQLYKRSENGSEKKKEILQQIKDAIKHRSHLDSSMQLIGDLLFGPKKASAILKS
VREPGSPLVDDWGCLKSMVRVFETCCGSLTQYGMKHMRTFANICNAGVSHTSMEEACNA
ACSGHDAGQWHPTNQGYSA

>XP_007012236.2 γVPE Theobroma cacao
MTRLVSGVILLLLSLTGIVSAGRDITGDVLRLPSEASKFFRGSNDDEVEGTRWAVLIAGSNG
YWNYRHQADVCHAYQLLKKGGLKDENIIVFMYDDIAFNEENPRPGIIINSPHGDDVYEGV
PKDYTGEDVTVNNLLAAILGNKTALTGGSGKVVDSGPNDHIFIYYTDHGGPGVLGMPTFP
YLYADDLIEVLKKKHASGTYKSLVFYLEACESGSIFEGLLPEGLNIYATTASNAEESSWGTY
CPGEYPSPPPEYETCLGDLYSVAWMEDSDIHNLQTETLHQQYELVKRRTINGNSAYGSHV
MQYGDIGLSKDIVFVYLGTNPANDNFTFVDENSLQPPTKAVNQRDADLVHFWDKYRKAP
DGSVRKLEAQKQFVEAMSHRMHIDNSMKLIGKLLFGIEKGPEVMKTVRPAGQPLVDDWK
CLKKMVRTFETHCGSLAQYGMKHMRSLANICNAGIQTEQMAEASAQACVSIPSGHWSSV
QKGFSA

>XP_017972845.1 βVPE Theobroma cacao
MAKQDSVFIKYLSLLLVLLLLFEAGRAARLNQWESGIRLSTDIDEPQDVDDQQLGTRWAV
LVAGSSGYANYRHQADVCHAYQLLRKGGLKEENIVVFMYDDIAMHKLNPRPGVIINHPK
GDDVYAGVPKDYTGVHVTAANLYAVLLGNKSALSGGSGKVVDSKPNDRIFLFYSDHGGP
GVLGMPNLPFLYAMDFLDVLKKKHAAGSYKEMVIYVEACESGSVFEGIMPKDLNIYVTT
ASNAQESSWGTYCPGMEPSPPPEYTTCLGDLYSVAWMEDSETHNLKRETVKQQYETVRE
RTSNFNSYTLGGSHVMEYGNASIKAEKLCSYQGFDPSSENFPPNELTHMEAVNQRDADILF
LWHMYKNSEDGSKKTEILRQITETIRHRIHLDGSIDLIGTLLYGPAKGSGVLNSVREPGLPL
VDDWQCLKSMVRLFETHCGLLTQYGMKHMRAFANLCNSGVSQSLMEQACVAACSGHD
TRQWHPSNQGYSA

>XP_002880728.1 αVPE Arabidopsis lyrata
MTTVAVTFLALFLYLVAAVSGDVIKLPSQASKFFHPTENDDDSTRWAVLVAGSSGYWNYR
HQADVCHAYQLLKKGGVKEENIVVFMYDDIAKNEENPRPGVIINSPNGEDVYNGVPKDY
TGDDVNVDNLLAVILGNKTAVKGGSGKVVDSGPNDHIFIYYSDHGGPGVLGMPTSPYLYA
NDLNDVLKKKHASGTYKSLVFYLEACESGSIFEGLLPEGLNIYATTASNAVESSWGTYCPG
EDPSPPSEYETCLGDLYSVAWMEDSDIHNLQTETLHQQYELVKKRTAGSGKSFGSHVMEF
GDIGLSKEKLVLYMGTNPANENFTFVNENSLRPPSRVTNQRDADLVHFWDKYRKAPEGSA
RKVEAQKQVLEAMSHRLHVDNSILLIGKLLFGLDSPAVLNNVRPSGTPLVDDWDCLKSLV
RVFEMHCGSLSQYGIKHMRSIANICNAGIQMGQMEEAAMQACPTIPASPWSSLERGFSA

>XP_002867219.1 γVPE Arabidopsis lyrata
MATTMTRVPVGAFLLVLLVSLVAVSTARSGPDDVIKLPSQASRFFRPAQDDDDSNAGTRWA

VLVAGSSGYWNYRHQADICHAYQLLRKGGLKEENIVVFMYDDIANNYENPRPGTLINSPH
GKDVYQGVPKDYTGDDVNVDNLFAVILGDKTAVKGGSGKVVDSGPNDHIFIFYSDHGGP
GVLGMPTSPYLYANDLNDVLKKKHASGTYKSLVFYLEACESGSIFEGLLPEGLNIYATTAS
NAEESSWGTYCPGEEPSPPPEYETCLGDLYSVAWMEDSGMHNLQTETLHQQYELVKRRT
APVGYSYGSHVMQYGDVGLSKDNLDLYMGTNPANDNFTFADANSLKPPSRVTNQRDAD
LVHFWEKYRKAPEGSARKTEAQKQVLEAMSHRLHVDNSVILVGKILFGISEGPEVLNKVR
SAGQPLVDDWNCLKNLVRAFERHCGSLSQYGIKHMRSFANICNAGIRTEQMEEAASQACT
SIPPGPWSSLHRGFSA

>XP_008796848.1 VPE Phoenix dactylifera
MATVTCSAIGWIALLSALLLPIGHCAGRVAGGWDSVIRMPAEMGSDDGDEEIGTRWAVLV
AGSFGYGNYRHQADVCHAYQLLKKGGLKEENIVVMMHDDIANNPLNPRPGVIINHPDGE
DVYAGVPKDYTGEQVTTKNLYAVILGNKSAVEGGSGKVVDSKPVDRIFIYYSDHGGPGVL
GMPNMPFLYAADFIDVLKKKHASGSYREMVIYVEACESGSIFEGLLPEGLNIYVTTASNAE
ESSWGTYCPGMDPAPPPEYITCLGDLYSVAWMEDSATHNLKEETIEKQYEVVKERTSNYN
TYSAGSHVMEYGDKSFKDDKLYLFQGFDPSNANLSGNGLPPTMPTGAINQRDADLLFLW
KRYEQLDARSEKKTQVLKEITETMMHRLHLDNSINLIGKLIFGSENGPSILNAVRPSGQAL
VDDWNCLKTMVQVFQSYCGPLTQHGMRHMRAFANLCNRGISNDAMVEACVNTCGSHIS
AKWSPSSREYSAF

>XP_010933722.1 VPE Elaeis guineensis
MATATSSAIGWIVLLSALVLPIGHSAFRVGGGWESVIRMPTESGGDDGEQEIGTRWAVLVA
GSFGYGNYRHQADVCHAYQLLKKGGLKEENIVVMMHDDIANNPLNPRPGVIINHPQGED
VYAGVPKDYTGEHVTTTNLYAVILGNKSAVEGGSGKVVDSKPNDRIFIYYSDHGGPGVLG
MPNMPFLYAADFIDVLKKKHASGGYKEMVIYVEACESGSIFEGLMPEDLNIYVTTASNAE
ESSWGTYCPGMDPSPPPEFITCLGDLYSVAWMEDSETHNLKEETIQKQYEEVKERTSNYNT
YNTGSHVMEYGDKSFKDDKLYLFQGFDPSNANLSGNALAPTMPMEAINQRDADLLFLW
KRYEQFDEGSKKKTEALREITETLVHRLHLDNSINLIGKLIFGSENGPSILNAVRPSGQALV
DDWNCLKTMVQVFQTYCGPLTQYGMRHMRAFANICNRGSSKDAMVEACLNACESHISA
KWSSSSQEYNA

>XP_009398114.2 VPE Musa acuminata subsp. Malaccensis
MAYMVRSFSLIGRLLFSSTLLWASLFATPGPRTVTAGRAVGQWDPTIRLPTERAGLDGLGG
GVDEKEQEDEETSGTRWALLVAGSSGYGNYRHQADVCHAYQLLRRGGLKEENIVVMMY
DDIANSPLNPRPGVIINHPQGHDVYAGVPKDYTGKQVTSKNLYAVLLGIKSAVTGGSGKVI
DSKPNDRIFIYYSDHGGPGVLGMPNMPYLYAVDFIEVLKKKHAMNSYKEMVIYVEACESG
SIFEGLMPKDLNIYVTTASNAEESSWGTYCPGMDPPPPPEYITCLGDLYSVAWMEDSETHN
LKEETVSKQYEAVKVRTSNYNTYSVGSHVMEYGDKNIKPEKLYLYQGFDPANANITENGL
SQRMQMGTINQRDADLLFLWKRYERLAESSEDKRRTVMEITETMMHRTHLDRSIDLIGKL
IFGSNSGPAILRAVRPYGQALVDDWDCLKSMVRSFESHCGSLTQYGMKHMRAFANICNRG
ISRDAIKEASASACGNYNSAMWSSSMRGYSA

>XP_006302155.1 VPE Capsella rubella
MAKSYYFRPALLLLLLLLLVRAESRGWFEPKILMPTEEAKPADQDEDEDGVGTRWAVLVA

GSSGYGNYRHQADVCHAYQILRKGGLKEENIVVMMYDDIANHQLNPRPGTLINHPNGED
VYAGVPKDYTGSNVTAANFYAVLLGDQKAVKGGSGKVIASKPNDHIFVYYADHGGPGVL
GMPNTPHIYAADFIETLKKKHASGTYKEMVIYVEACESGSIFEGIMPKDLNIYVTTASNAQ
ESSYGTYCPGMNPSPPSEYITCLGDLYSVAWMEDSENHNLKKETIKQQYQTVKMRTSNYN
TYSAGSHVMEYGNDSIKAEKLYLYQGFDPATVSLPPNELPVKSQVGVVNQRDADLLFLW
HMYRRSEDGSRKKDDTLKELTETTRHRKHLDASVELIDTILFGPAMNVLNSIREPGLPLVD
DWECLKSTVRVFETHCGSLTQYGMKHMRAFANVCNNGISKELMEEASTAACGGYIEARY
MLHPSILDYSA

>XP_004515742.1 VPE Cicer arietinum
MAVDSKKMCSKNVLSSWPLILLLLSFHGTAARLNRFEWDSVIRLPTEPVDADSDEGGTRW
AVLVAGSSGYGNYRHQADVCHAYQLLVKGGVKEENIVVFMYDDIAQNELNPRPGVIINHP
QGPDVYAGVPKDYTGDSVTAENLYAVILGDKSKVKGGSGKVINSKAEDRIFIYYSDHGGP
GVLGMPNMPYLYAMDFINVLKKKHASRGYKKMVIYVEACESGSIFEGIMPNNLNIYVTTA
SNAQENSWGTYCPGLDPAPPPEFITCLGDLYSVAWMEDSETHNLKRETVKEQYKSVKERT
SNSNNYALGSHVMQYGDTNITDEKLYLYHGFNPATVNFPPHNGRLETKMEVVNQRDAEL
FFMWQLYKRLDHEAEKKRDILEKIAETVKHRNHLDGSVELVGVLLFGPEKGSSVLQSVRA
PGLPLVDDWECLKSRVRVFETHCGSLTQYGMKHMRSFANICNNDISETSFEEACLKACGG
YDVGLLHPSNGGYSA

>XP_006440379.1 VPE Citrus clementine
MLFKYDFLRSYHLTLPLFVLFLDCFFITNFLNFFHALSMATAHHRSVFFLLVLVLVLFTGGV
QAARFNRRDSAILLPSEKQEPPAEEGGEPVGTRWAVLVAGSSGYANYRHQADVCHAYQLL
RKGGLKEEHIVVFMYDDIAMHELNPRPGVIINHPQGENLYDGVPKDYTGEHVTAQNLYAV
LLGDRKAVKGGSGKVVNSKANDRIFIFYSDHGGPGVLGMPNMPYVYAMEFIDVLKKKHA
AKSYKGMVIYVEACESGSIFEGVMPKDLDIYVTTASNAQESSFGTYCPGMDPSPPPEYITC
LGDLYSVAWMEDSETHNLKRETISQQYQAVKERTSNFNNYNSGSHVMEYGNTSVKSEKL
YLYQGFDPASTNFPPNKLQPDQMGVVNQRDADLLFMWHMYKNAAEGSEKKSEMLKQIT
ETMRHRKHLDASIDMIGVILFGPDKGSRILNSVGARGLPLVDDWQCLKSMVRVFETHCGS
LTQYGMKHMRAFANICNSGVSQALMEETSEAACSGNELRQWHPAIRGYSA

>XP_006477253.1 VPE Citrus sinensis
MLFKYDFLHSYHLTLSVFVLFLDCFFITNFLNFFHALSMATAHHRSVFFLLVLVLVLFTGGV
QAARFNQRDSAILLPSEKQEPPAEEGGEPVGTRWAVLVAGSSGYANYRHQADVCHAYQLL
RKGGLKEEHIVVFMYDDIAMHELNPRPGVIINHPQGENLYDGVPKDYTGEHVTAQNLYAV
LLGDRKAVKGGSGKVVNSKANERIFIFYSDHGGPGVLGMPNMPYVYAMEFIDVLKKKHA
AKSYKEMVIYVEACESGSIFEGVMPKDLDIYVTTASNAQESSFGTYCPGMDPSPPPEYITC
LGDLYSVAWMEDSETHNLKRETINQQYQAVKERTSNFNNYNSGSHVMEYGNTSVKSEKL
YLYQGFDPASANFPPNKLLPDQMGVVNQRDADLLFMWHMYKNAAEGSEKKSEMLKQIT
ETMRHRKHLDASIDMIGVILFGPDKGSRILNSVRARGLPLVDDWQCLKSMVRVFETHCGS
LTQYGMKHMRAFANICNSGVSQALMEETSEAACSGNELRQWHLAIRGYSA

>XP_004298668.1 VPE Fragaria vesca
MAARLSKLLLLFVALVIQTTIGGGGRAAARLDFWDSAAIRLPSEKDKTEDAVEQSGTTWA

VLVAGSNGYGNYRHQADVCHAYQILKKGGLKEENIVVFMYDDIAMHEMNPRKGIIINHP
QGQDVYAGVPKDYTGEHVNAANLYAVLLGDKKAVKGGTGKVVASKPNDRIFLFYSDHG
GPGVLGMPNMPFLYAMDFINVLKKKHASGSYKEMVIYVEACESGSIFEGIMPSDLNIYVAT
ASNAEENSFGTYCPGMNPPPPPEYITCLGDLYSVAWMEDSERHNLKKETIKEQYQTVKER
TSNFNNYNIGSHVMQYGSKNLTEEKLYLYLGFDPASVNFPPNNGQLEQHMEVVNQRDAEI
FFMWQLYKRSEHGSEKKREILKQIRDTMNHRNHIDGSIKFIGTFLYGPKNGATVLNSVRPL
GLPLVDDWECLKSMVRVFETHCGSLTQYGMKHMRAFANICNSGVSQAQMEEASWAACD
GHDLGHLHPSNKGYSA

>XP_006647602.1 VPE Oryza brachyantha
MAAQWCFALLLALSAAAEAGAKRMWEPVIRMPGEVVVEEEVVGGIGTRWAVLVAGSSG
YGNYRHQADVCHAYQILRKGGLKEENIVVFMYDDIANNALNPRPGVIVNHPQGEDVYAG
VPKDYTGDEVNTKNFYAVLLGNKTAVTGGSRKVIDSKPNDHIFIFYSDHGGPGVLGMPNL
PYLYAADFMKVLQEKHVSNTYAKMVIYVEACESGSIFEGLMPEDLNVYVTTASNAEESS
WGTYCPGMEPSPPAEYITCLGDLYSVSWMEDSETHNLKEESIKEQYEVVKKRTSDMNSY
GAGSHVMEYGDKTFKGEKLYLYQGFDPANAEVKNKLLLDDPKAAVNQRDADLLFLWRR
YELLHEKSEEKLKVLREISEAVTHRKHLDSSVDFVGKLLFGFGNGPTVLQHVRPSGQPLV
DDWDCLKRMVRIFESHCGSLTQYGMKHMRAFANICNNGIPDAAMKDASIVACSSDNSAR
WSSLVQGYSA

> XP_004953273.1 βVPE Setaria italica
MAVCRCLVVLVAALAAAAAGAGTEEGDWDPVIRMPGEEEPAAARGGEPLDEEEDDGVG
TRWAVLVAGSSGYGNYRHQADICHAYQILLKGGLKEENIVVFMYDDVANSVLNPRQGVII
NHPEGEDVYAGVPKDYTGEQVTAKNFYAVLLGNKDAVTGGSRKVINSKPNDHIFIYYSDH
GGPGVLGMPNLPYIYAGDFMKVLREKHASNSYAKMVIYVEACESGSIFEGLMPEDLNIYV
TTASNAEESSWGTYCPGMIPSPPSEYITCLGDLYSVSWMEDSETHNLKEETIKEQYEAVKE
RTSDSNSYGAGSHVMEYGDKTFKGEKLYLYQGFNPENADITYKLLWQGQKSVVNQRDA
DILFLWKRYELLNEKSKEKLEVLREITGTVTHRKHLDSSVDFIGKLLFGVENGPSTLGAVR
SPGQPLVDDWDCLKRMVRIFESHCGSLTQYGMKHMRAFANICNRGTPATAMKEASISACG
SYNSARWSPLVQGYSA

>XP_004976374.1 VPE Setaria italica
MAAAAWLCGLLSLLAVAAAASVDGAEEEWEPLIRMPTEKGGNAAAAAPAAEEDEVGTR
WAVLVAGSSGYGNYRHQADVCHAYQILLKGGVKEENIVVFMYDDIAHNILNPRPGVIINH
PKGENVYPGVPKDYTGDQVTTENFFAVLLGNRSAITGGSKKVIDSKPNDHIFIYYSDHGGP
GVLGMPNLPYLYAGDFIKVLKKKHASNSYSKMVIYVEACESGSIFEGLMPQDLNIYVTTA
SNPVENSWGTYCPGMDPSPPPEYITCLGDLYSVSWMEDSQTHNLMKETIKDQYEVVKTR
TSNLKKYKEGSHVMEYGDKTFTNEKLFLYQGFDPANANAANTLLWPGPKGAVNQRDAD
LLFMWKRYEQLDGGSEEKLRALREIKETVQHRKHLDSSIDFIGRLVFGFENGPKMLEAVR
ASGQPLVDDWDCLKRMVRIFEAQCGSLTQYGMKYMRAFANICNSGISEAKMRESSISACG
GYNSARWSPMAQGHSA

>Peaxi162Scf00002g00516.1 Petunia axillaris
MGSFSFAVTCMILMLLVMVVAIPFEPKIGKRISRLHHRWDPLIRSPVDRDDETEDNDGVRW

AVLVAGSQGYGNYRHQADVCHAYQILKRGGLKDENIVVFMYDDIAKSELNPRHGVIINHP
NGSDVYAGVPKDYTGEHVTVANLYAVLLGDKSAVKGGSGKVVDSKPNDRIFLYYSDHGG
PGVLGMPNMPFLYGKDLIEVLKKKHASGTYKEMVLYIEACESGSVFEGLMPEDLNIYVTT
ASNAEESSWGTYCPGMDPPPPPEYITCLGDLYSVSWMEDSESHNLKKETLEQQYKKVKE
RTSNFNNYNAGSHVMEYGSKDIKSEKVYLYQGFDPATVNLPANKIDFTHLDVVNQRDAD
LLFLWERYKKLADNSLEKAKLKKEITETMLHRQHLDGSVDAIGLFLFGPTKGSSVLNSVR
GPGLPLVDDWDCLKSTVRLFEAHCGSLTQYGMKHMRAFANICNDGVSRDAMEEAFMAA
CSEHKIEEYSPANRGFSA

>Peaxi162Scf00436g00515.1 Petunia axillaris
MIRYVATTLFLIGLSLNIFVSESRNVLRLPSEVSRFFGADESVRNKDDDSVGTRWAILLAGS
NGYWNYRHQADICHAYQLLKKGGLKDENIVVFMYDDIANNEENPRPGIIINSPHGEDVYK
GVPKDYTGDDVTVDNFLAVILGNKAALSGGSGKVVNSGPNDHIFIYYSDHGGPGVLGMP
TDPYLYANDLIDVLKKKHASGTYKSLVFYLEACESGSIFEGLLPEGLNIYATTASNAEESSW
GTYCPGEYPSPPIEYETCLGDLYSIAWMEDSDIHNLRTESLKQQYHLVKDRTANGNPFYGS
HVMQYGDLHLSKNPLFVYMGTNPANDNYTFGADNSLRVSKVVNQRDADLLHFWYKFR
KAPEGSARKFEAQKQLNEAISHRMHLDNSIALVGKLLFGIKNVPEVLSSVRPAGQPLVDD
WDCLKSYVRTFETHCGSLSQYGMKHMRSIANICNAGIKMEQMVEASAQACPRVPSNTWS
SLHRGFSA

>Peaxi162Scf00972g00037.1 Petunia axillaris
MISHVAGILILVGFSILGAGEGRNVLKLPSEASKFFKKGEDDDSVGTRWAADVCHAYQLLR
KGGLKDENIVVLMYDDIAYNEENPRKGVIINNPAGEDVYKGVPKDYTGDDVNVDNFLAV
LLGNKTAITGGSGKVVDSGPNDHIFIFYTDHGGPGVLGMPTKPYLYASDLIGALKKKHAS
GTYKSLVLYIEACEAGSIFEGLLPEGLNVYATTASDAVEGSWITYCPGQNPSPPPEYTTCLG
DLYSVSWMEDSEKHNLQTESLRQQYHLVKRKIAYASHVMQYGDLKLSMDSLSMYMGTD
PANDNYTFVDDNSLGASSKAVNQRDADLLHFSEKFLKAPEGSARKVEAQKQFAEAMSHR
MHLDSSMALVGKLLFGIQKGPEVLKRVRSDGQLLVDDWACLKSFVRTFETHCGSLSQYG
MKHMRSFANICNAGIEVEQMVEASSQACPSVPSNTWSSLHRGFSA

>Peaxi162Scf00064g01633.1 Petunia axillaris
MINVAGILILVGFSIIAAGEGRNVLKLPSEASRFFDKGDDDSADVCHAYQLLRKGGLKDEN
IIVFMYDDIAYNEENPRKGVIINSPAGEDVYKGVPKDYTGDDVNVDNFLAVLLGNKTALT
GGSGKVVDSGPNDHIFVFYSDHGGPGVLGMPTNPYLYASDLIGALKKKHASGTYKSLVLY
IEACESGSIFEGLLPEGLNVYATTASNAVESSWGTYCPGENPSPPPEYETCLGDLYAVSWME
DRTANGNSAYGSHVMQFGDLKLSVDSLSMYMGTDPANDNSTFVDDNSLGASSKAVNQR
DADLLHFWDKFLKAPEGSARKVEAQKQFTEAMSHRMHLDNSMALVGKLLFGIQKGPEV
LKRVRSDGQPLVDDWACLKSFVRTFETHCGSLSQYGMKHMRSIANICNAGIKMEQMVEA
SSQACPSIPSNTWSSLHRGFSA

>Solyc08g079160.2 Solanum lycopersicum
MGSCNFTVCVTLMLLVMVGAISIEPKIDSRRLGRPHRFWDPLIRSPVDRDDDDETEEGGG
GVRWAVLVAGSNGYGNYRHQADVCHAYQILKRGGLNDENIVVFMYDDIAKSELNPRPGV
IINHPNGSDVYAGVPKDYTGEHVTAANLYAVLLGDKSAVKGGSGKVVNSGPNDRIFLYYS

DHGGPGVLGMPNMPYLYGKDLIEVLKKKYAARTYKEMVLYIEACESGSVFEGLMPENLN
IYVMTASNAEESSWGTYCPGMDPPPPSEYITCLGDLYSVAWMEDSESHNLKKETIKQQYE
KVKERTSNSNNYNAGSHVMEYGSKEIKPEKVYLYQGFDPATVNLPANKIDFARLEVVNQR
DADLLFLWERYKKLEDNSLEKAKLRKEITETMLHRQHLDGSIDAVGVFLFGPIKGGSVLSS
VRKPGLPLVDDWECLKSTVRLFEAHCGSLTQYGMKHMRAFANICNNGISSDAMEDAFMA
ACNGHSLEEYTTANRGFSA

>SGN-U578962 Solanum lycopersicum
MFVKINVASFLIALFVVLTEGRNVIERFDEDYEDSIGTKWAVLVAGSKEWYNYRHQANLC
HAYQLLKKGGLKDEHIIVFMYDDIANNPENPRPGVIINNPHGHDVYKGVPKDYTGKDCN
AQNFYSVILGNKSALTGGSGKVVNSGPNDYIFIYYTDHGAPGLVGMPEDPPVYAIDLNEVL
KKKHASRTYKKMVFYLEACDSGSMFADLLDEGLNIYATTSSKPDEDGWATYCYFTGDTS
CYGECPPKDFKDNCLGDLFSVSWLENSDLHDLQVETLEKQYLRIHKRVLNNGTHGSHMM
QYGDLHINKDALSIYMGSNSPKHTSSANNNNASNSRHVNQRDVQLLYLISKFQNAPEGSR
RKNEAYRKSEVISEREHVDKSVKHIGQILFGVENGQKVLNIVRQPLVDDWHCLKSFVKIFE
SHCGSLTSYGKKHIRGFANMCNAGIQRDQMDAAAKQTCSS

>SGN-U580226 Solanum lycopersicum
MMESIFEDHENSIGTKWAVLVPGPNEWYNYRHQADICHAYQLLKKGGLKDENIVVFMYD
DIAYNSENPKPGVIINKPHGPDVYKGVPKDYTGKHCNAQNFYGVVLGNKSALTGGSGKV
VNSGPNDYIFIYYADHGGSGVIDMPIEPSIYAKDLNEVLKKKHASRTYEKMVFYLEACESG
SMFEGLPYKGLNIYVTTASKADENSYATYCSPKGYESTCLGDLFSVSWLENSELQDRQVE
TLKKQYQRIRKRVLNNGTEGSHMMEYGDLHIHEDALSIYMGSNFPTHTSSTKNNYALKLE
TC

>SGN-U580215 Solanum lycopersicum
MNRSVAGVLFLIALSLNVSVSESRNFLKLPSEGSRFFDADEIDSVGTRWAILLAGSNGYWN
YRHQADICHAYQLLKKGGLKDENIVVFMYDDIANNEENPRQGVIINSPHGEDVYNGVPK
DYTGDDVTVDNFLAALLGNKTALTGGSGKVVDSGPNDHIFIFYSDHGGAGVLGMPTNPY
LYANDLIDALKMKHASGTYKSLVFYLEACESGSMFEGLLPEGLNIYATTASNADESSWGT
YCPGEYPSPPIEYDTCLGDLYSISWMEDSERHNLRTESLKQQYHLVKERTASGNPAYGSHV
MQYGDVHLSKDAVFLYMGTDPANDNSTFMDDNSLRVSKAVNQRDADLVHFWYKFHKAP
EGSVSKTEAQKRLNEAISHRMHLDNSIALVGKLLFGIKKGPEVLTSVRPAGQPLVDNWDC
LKSYVRTFETHCGSLSQYGMKHMRSVANICNAGIKMEQMVEASAQACPSVPSYTWSSLH
RGFSA

>SGN-U567075 Solanum lycopersicum
MMGSCNFTVCVTLMLLVMVGAISIEPKIDSRRLGRPHRFWDPLIRSPVDRDDDDETEEGG
GGVRWAVLVAGSNGYGNYRHQADVCHAYQILKRGGLNDENIVVFMYDDIAKSELNPRPG
VIINHPNGSDVYAGVPKDYTGEHVTAANLYAVLLGDKSAVKGGSGKVVNSGPNDRIFLYY
SDHGGPGVLGMPNMPYLYGKDLIEVLKKKYAARTYKEMVLYIEACESGSVFEGLMPENL
NIYVMTASNAEESSWGTYCPGMDPPPPSEYITCLGDLYSVAWMEDSESHNLKKETIKQQY
EKVKERTSNSNNYNAGSHVMEYGSKEIKPEKVYLYQGFDPATVNLPANKIDFARLEVVNQ
RDADLLFLWERYKNLEDNSLEKAKLRKEITETMLHRQHLDGSIDAVGVFLFGPIKGGSVL

SSVRKPGLPLVDDWECLKSTVRLFEAHCGSLTQYGMKHMRAFANICNNGISSDAMEDAF
MAACNGHSLEEYTTANRGFSA

>SGN-U579062 Solanum lycopersicum
MVHVAGVFILVGIAVLAAVEGRNVLKLPSEASRFFDDADDSVGTRWAVLLAGSNGYWNY
RHQADVCHAYQLLRKGGLKDENIIVFMYDDIAHHEENPRPGVIINSPAGEDVYEGVPKDY
TGDDVNVHNFLAVLLGNKTALTGGSGKVVNSGPNDHIFIFYSDHGGPGVLGMPTNPYLYA
DDLIAVLKKKHAAGTYKSLVLYIEACESGSIFEGLLPNGLNIYATTASNAEESSWGTYCPGE
YPSPPPEYETCLGDLYAVSWMEDSEMHNLRTENLRQQYHLVKKRTANGNTAYGSHVMQF
GDLQLSMESLFRFMGTNPANDNYTYVDDNSLLASSKAVNQRDADLLHFWDKFRKAPEG
SARKVEAQKQFTEAMSHRMHLDERIALVGKLLFGIQKGPEVLKHVRSAGQPLVDDWACL
KSFVRTFESHCGSLSQYGMKHMRSIANICNAGIQMEQMVEASAQACPSIPSNIWSSLHRGF
SA

>XP_002448237.1 Sorghum bicolor
MAAAAWLCGLLWLLAHAAAVASAADGADGGWEPLIRMPTGKGGDAAARAVEEDDEVG
TRWAVLVAGSSGYGNYRHQADVCHAYQILRKGGVKEENIVVFMYDDIAHNILNPRPGVII
NHPKGENVYNGVPKDYTGDQVTTENFFAVLLGNKSAITGGSKKVIDSKPNDHIFIYYSDH
GGPGVLGMPNLPYLYAGDFIKVLKKKHACNSYSKMVIYVEACESGSIFEGLMPEDLNIYV
TTASNPVENSWGTYCPGMEPSPPPEYITCLGDLYSVSWMEDSQTHNLKKETIKDQYEVVK
TRTSNSNKYKEGSHVMEYGDKTFKDEKLFLYQGFDPANANIANMLLWPGPKGAVNQRD
ADLLFMWKRYEQLNGESVEKLRALIEIKETVQHRKHLDSSIDFIGRLLFGFEKGPSMLEAV
RASGLPLVDDWDCLKRMVRIFESQCGSLTQYGMKYMRAFANICNSGISEMKMRESSISAC
SSYNSARWSPMAQGHSA

>XP_002452849.1 Sorghum bicolor
MAARWCLVLLVLVLAAAAAGAEKGEWDPVIRMPGEEEPAASSHSHSGEGFEGEEDVAVG
TRWAVLVAGSSGYGNYRHQADICHAYQILRKGGIKEENIVVFMYDDVATSALNPRQGVIIN
HPQGEDVYAGVPKDYTGDQVTAKNFFAVLLGNKTAVTGGSRKVINSKPDDHIFICYSDHG
GPGVLGMPNLPYLYAGDFMKVLREKHASNSYAKMVIYIEACESGSIFEGLMPEDLNIYVTT
ASNAEESSWGTYCPGMEPSPPSEYITCLGDLYSVSWMEDSETHNLKEETIKEQYEVVKER
TSDSNSYGAGSHVMEYGDKTFKGEKLYLYQGFDPANANVTNKLLRPGLEAVVNQRDADI
LFLWKRYELLHEKSEEKQEVLREITGTVRHRKHLDSSIDFIGKLLFGIEKGPFTLQAVRPSG
QPLVDDWDCLKQMVRIFESHCGSLTQYGMKHMRAFANICNSGTPGASMKQASMGACGS
YNSARWSPLVQGYSA

>Csa011434 Cucumis sativus
MARIPTGVLLSLLFLAVIGLPAGARDLPGDFLRLPSEALKFFRGGASDASDEDSVGTRWAV
LIAGSNGYWNYRHQADICHAYQLLRKNGLKDENIIVFMYDDIAFNPENPRPGVIINHPKGS
DVYHGVPKDYTGEDVTVNNFFAAILGNKTALTGGSGKVVDSGPNDHIFIYYSDHGGPGVL
GMPTYPYMYADDLNKVLKKKHAAGSYKSLVFYLEACESGSIFEGLLPEGLNIYTTTASNA
YESSWGTYCPGDYPSPPPEYDTCLGDLYSVAWLEDSDNHNLKTESLRQQYELVKKRTLSG
QYAYGSHVMQYGDLMLNKNALFSYLGTDPANENNTFVEENSLRPATKFTNQRDADLVHF
WEKFRKAPE GSLTKVEAQKKFVEAMSHRAHIDNSVKLVGKLLFGIKEGPEVLEAIRPAG

RPLVDDWNCLRNMVRSFEARCGSLSQYGMKHMRSFANLCNAGISKEQMAEASAQACMS
VPPGPWSSLLKGFTA

>Csa017587 Cucumis sativus
MASPSTSNLVLFLLLFLAYGCADASPWDRWERTIRMPTEKEEMGGAGDRKVGTRWAVLI
AGSSGFGNYRHQADICHAYQLLKKGGLKDENIVVFMYDDIATNVLNPRPGIIINHPQGED
VYAGVPKDYTGEHVTAQNLYAVLLGNRTAVDGGSGKVVDSKPNDRIFVYYSDHGGPGVL
GMPNLPFVYAMDFIEVLKKKHAAKGYKEMVIYVEACESGSIFEGILPKDLNIYVTTASNA
QESSFGTYCPGMEPAPPPEYMTCLGDLYSVAWMEDSETHNLKRETIDQQYRTVKERTSNP
NNLNTGSHVMEYGNSSIKAERLYLYQGFDPASVNLPPYNGRYEMKSMDAINQRDADIFFL
WQMYRKFEDGTNERAQVLEEIRETVTHRTHLDGSIRMIGFLLFGPEKGSNILDDVRASGLP
LVDDWECLKSMVRVLESYCGSLTQYGMKHMRAIANICNRGVSKASMREASMVACNGGS
YGLWHPSNRGYSA

>CA01g04690 Capsicum annuum
MGSCNFTVCVTLMLLVMVGAISIEPKIDSRRLGRPHRFWDPLIRSPVDRDDDDETEEGGG
GVRWAVLVAGSNGYGNYRHQADVCHAYQILKRGGLNDENIVVFMYDDIAKSELNPRPGV
IINHPNGSDVYAGVPKDYTGEHVTAANLYAVLLGDKSAVKGGSGKVVNSGPNDRIFLYYS
DHGGPGVLGMPNMPYLYGKDLIEVLKKKYAARTYKEMVLYIEACESGSVFEGLMPENLN
IYVMTASNAEESSWGTYCPGMDPPPPSEYITCLGDLYSVAWMEDSESHNLKKETIKQQYE
KVKERTSNSNNYNAGSHVMEYGSKEIKPEKVYLYQGFDPATVNLPANKIDFARLEVVNQR
DADLLFLWERYKKLEDNSLEKAKLRKEITETMLHRQHLDGSIDAVGVFLFGPIKGGSVLSS
VRKPGLPLVDDWECLKSTVRLFEAHCGSLTQYGMKHMRAFANICNNGISSDAMEDAFMA
ACNGHSLEEYTTANRGFSA

>CA12g18500 Capsicum annuum
MNDQVAGILFIVGLSVTVAVAVTVVDGRNVLKLPTEASRFFDHADDDSVGTRWAVLLAGS
NGYWNYRHQADVCHAYQLLRKGGLKDENIIVFMYDDIAYNEENPRPGVIINNPAAEDVY
EGVPKDYTRDDVNVHNFLAVLLGNKTALTGGSRKVVNSGPNDHIFIFYSDHGGPGVLGM
PTNPYLYASDLINALKKKHAAGAYKSLVLYIEACESGSIFEGLLPTGLNIYATTASNAVESS
WGTYCPGEYPSPPPEYETCLGDLYAVSWMEDSEMHNLRTENLKQQYHLVKRRTANGNTY
GSHVMQFGDLQLSMESLFSFMGTNPANDNYTYIDDNSLWASSRAVNQRDADLLHFWDKF
RKAPEGSARKVEAQKQFTEAMSHRMHLDNSIALVGKLLFGIQKGPEVLKRVRSAGQPLV
DDWACLKSFVRTFETHCGSLSQYGMKHMRSIANICNAGIHTEQMVEASAQACPSIPANTW
SSLHRGFSA

>CA02g06710 Capsicum annuum
MIFRYNICIVLLVVLSIWASIEGRSISRFLTDETVGTKWAVLVAGSHGWWNYRHQADVCHA
YQILKKGGLKDENIIVFMYDDIANNTMNPRPGVIINNPHGQNVYKGVPKDYVGKDVSAD
NFFNVILANKSGITGGSGKVLKSGPNDHIFIYYVDHGGPGILSMPTGFMYAKDLTDVLKKK
HASGTYNKLVFYLESCESGSMFDGLLPKGLNIYVTTASKPNEDSWGTYCGQVSASDPCLV
ECSPPEFKGLCLGDLNSVAWMEDSEAQDRRTATLGDQYNVIAKRTAANLAYGSHVMQYG
DTGISVDALFQYMGAASVNHSYVSMNFKSSSQNVNQRDAELFYLKSKYEDAPEGSDEHF
KTRAELTKTMAHRSQVDRSIEHLAELLFGAEKGNELLKTVRPAGQPLVDNWDCLKFYVK

TFEAQCGKLTSYGKKHIRGIANICNAGIESEQMTAATAQACPI

>CA03g09280 Capsicum annuum
MIFRYNIGIIVLVFVLSIWASVEGRSISKFVTEETVGTKWAVLVAGSNGWWNYRHQADVCH
AYQLLKKGGLKDENIIVFMYDDIANNTMNPRPGVIINNPRGQDVYNGVPKDYVGDDVNA
DNFFNVILANKSGITGGSGKVLNSSPNDHIFIYYTDHGGPGIVSMPTGVVYANDLINVLKK
KHASGTYSKLVFYLEACESGSMFDGLLPEGLSIYVTTASKPNENSWGTYCGVGDARDPCL
VECPPPEFKGVCLGDLYSVAWMEDSDVQSRQTETLDEQYHKIANRTAANLTYGSHVMQY
GDMELSFDALFQYMGVASKNHTYVSMDSKSFSVYSRNVDQRETEFFYWQSKYRDAPEG
SDEHFEARARLIEIVAHKSQVDNSVKHLGEILFGVEYGRETLQSVRPVGQPLVDNWDCLK
SYVAKFEAHCGKLTSYGKKHIRGIANICNAGIESEQMVAATLQACPP

>CA06g24000 Capsicum annuum
MISRYNIVGVFSLFWLSILVNIEGLSISQFLTEESEGTKWAVLVAGSNGWDNYRHQADVCH
AYQLLKDGGLKDENIIVFMYDDIASNRENPRPGVIINNPRGPDVYKGVPKDYIQEDVNAN
NFYNVILANKSAVVGGSGKVLNSDPNDHIFIYYTDHGGPGIVSMPSGEYVFANDLVDVLK
KKHSSGTYDRMVFYLEACESGSMFDGLLPEGLDIYAMTASEPDEDSWATYCGEGTPDDPC
LVACPPREFQGVCLGDLFSVAWMEDSDAQDREADSVQGQYSRVANRTAANITYADYGSH
VTEYGDTVVSFDSLAAYMGENSKNHSHASVDAMSLSRNVDQFSTELFYLFTKHQNAPEG
SDEKYEAHVKLKEVISQRTQVDNNVKHLGELLFGVEKGNEVLHSVRPAGQPLVDNWDCL
KSYVKIFESHCGRLTPYGRKHVRGIANICNAGIKSEQMAAASAQACSI

>Achn093291 Actinidia chinensis Planch
MEANRFEFTSIVALLLLLVAPALGAGRTGRKNRWDQVIRMPGEADGPEEKGTRWAVLVAG
SQGYGNYRHQADVCHAYQILKRGGLKDENIVVFMYDDIASNVLNPRPGVIINHPQGQDV
YAGVPKDYTGQYVTSENLYAVLLGDKKDVKGGSGKVVDSKPNDRIFIYYSDHGGPGVLG
MPNMPFLYANEFIEVLKKKHASGSYKEMVIYVEACESGSVFEGMMPEDLNVYVTTASNA
EESSWGTYCPGMDPAPPAEYITCLGDLYSVAWMEDSESHNLKKETIAQQYTKVKERTSNY
NTYNAGSHVMEYGNKSIKSETLYLYQGFDPATENLPPNKIHLDTRMDVINQRDADLLSLW
QRYKRSEEGSEKKTKILKQITETMKHRIHLDSSIDMIGVLLFGPKKGHSILSSMRKPGSAIV
DDWTCLKSMVQVFETHCGSLTEYGMKHMRAFANICNNGVSGAAMEEACIVACGGHNSG
ELHPSKRGYSA

>MDP0000122571 Malusdomestica
MAVQHQSKLMISNYTVSWISLFLFVLLIQAIGGGRAARLERWDSGIRLPSEKDKPEDVHH
KEPGTRWAVLVAGSNGYGNYRHQADICHAYQLLKKGGLKEENIVVFMYDDIAKHLMNPR
PGVIINHPQGGDVYAGVPKGFASLTFFPSFLCSLYNTTIRVGEDQVAMVDYTGEQVTAANL
YAVLLGDKKAVKGGSGKVVDSKPNDRIFLYYSDHGGPGVLGMPNMPFLYAMDFIEVLKK
KHASGSYKEMVIYIEACESGSIFEGIMPKDLNIYVTTASNAQENSFGTYCPGMDPPPPPEYI
TCLGDLYSVSWMEDSERHNLKKETIEQQYLVVKQRTSNSNNYDVGSHVMEYGSKNITQE
KLYLYLGFDPASVNLPPNNGQLEKPMEVVNQRDAEIFYMWQMYKRSEHGSGKSREILNEI
KETMRHRTHLDASIQFIGTFLYGPGKGPSKLNSVRAVGQPLVDDWGCLKSMLQDLIPRLN
FELQVRVFETHCGSLTQYGMKHMRAFANICNSGVSEAKMAEACSAACNGHEVGQLHPSN
KGYSA

>MDP0000248773 Malusdomestica
MTRLASAVVLLFFASVLASAAGSRDLIGDVLRLPSEASKFFGRGDDAPDQQDDGTVGTRW
AVLIAGSNGYWNYRHQADICHAYQLLKKGGLKDENIVVFMYDDIAYNEENPRQGVIINSP
HGSDVYEGVPKDYTGEDVTVNNFFAAILGNKTALTGGSGKVVDSGPNDHIFIYYTDHGGP
GILGMPTSPYIYANDLIEVLKKKHAAGTYKSLVFYLEACESGSIFEGLLPEGLNIFATTASNA
EESSWGTYCPGEYPSPPPEYDTCLGDLYSVAWMEDSDVHNLRSETLHQQYELVKMRTAN
DNSGFGSHVMQYGDVGLSKNNLFVYMGTNPANDNYTFLGENSLRPSSKAVNQRDADLL
HFWHKYRKAPEGSARKIQAQKDFVEAMSHRMHIDQTMKLIGKLLFGIEKGPQVLNAVRP
AGQPLVDDWDCLKTMVRSFETHCGSLSQYGMKHMRSLANICNAGMTQEQMAEASAQA
CVSAPSGRWSSLHRGFSA

>MDP0000084203 Malusdomestica
MTRLASALVLLFLASVSASAAGSRDLNGNVLRLPSEASRFFGRGDNAPDQDDGTVGTRW
AVLIAGSNGYWNYRHQADICHAYQLLKKGGLKDENIVVFMYDDIAYNEENPRQGVIINSP
HGSDVYKGVPKDYTGQDVTVNNFFAAILGNKSALTGGSGKVVDSGPNDHIFIYYSDHGG
PGVLGMPATSPYIYANDLIEVLKKKHAAGTYKSMVFYLEACESGSIFEGLLPEGLNIFATTA
SNAEESSWGTYCPGEYPSPPPEYETCLGDLYSVAWMEDSDIHNLKSETLHQQYELVKTRT
ANDNSGFGSHVMQYGDVGLSKNNLFVYMGTNPANENFTFLGQNSLRPSSKAVNQRDAD
LVHFWHKYSKAPEGSPRKVQAQKEFVEAMSHRMHIDETMKLIGKLLFGIEKGPXVLNAV
RPAGQPLVDDWDCLKTMVRSFETHCGSLSQYGMKHMRSLANICNAGMTKEQMAEASA
QACVSAPSXRWSHMGFSA

>MDP0000321943 Malusdomestica
MVIVGVLLSLTLXSLAIHGSFCFPEINGDNKGSPRTTTDKGKRWAVLVAGSSGYDNYRHQA
DICHAYQILKKGGLKDENIIVFMYDDIAYNSENPRKGVIINKPNGHDVYKGVPKDYTGDH
VNARNLYAVILGDKSALTGGSGKVLSSGPNDHVFIYYADHGSVGLLGMPSDYVYAKDLIR
VLQKKHASKGYKSMVFYIEACEAGSMFEGLLSSNLNIYATTASNAEESSYGTYCPGDPSVP
EEFDTCLGDLYSISWMEDCSDISDLHKETLENQYETVRRRTTNSHVMQYGDMSHKQEFLF
AYMGTDLSNRSHTSTSDISSPSISRAVDQRDTKLLYFQQKLXRAPTGSQEKQGAQKQLLLE
IAHRKXVDYSITKLGEXLFGHEKSSNVLMNVRPQGQPVVDNWDCFKNFXNIYEKYCGHL
SAYGMKYTRAIANXCNAGITTEKMVAASDQTCANKPNV

>MDP0000166283 Malusdomestica
MANHGYCGVLLSLTLFSLAIHGSFCFPEINGXNKGSPRTTTDKGKXWAVLVAGSSGYDNY
RHQADICHAYQILKKGGLKDENIIVFMYDDIAYNSENPRKGVIINKPNGHDVYKGVPKDY
TGDHVNARNLYAVILGDKSALTGGSGKVLSSGPNDHVFIYYADHGSVGLLGMPSDYVYAE
DLIRVLKKKNASKGYKSMVFYIEACEAGSMFEGLLSSNLNIYATTASNAEESSYGTYCPG
DQSVPAEFDTCLGDLYSISWMEDCDISDLHKETLENQYERVRRRTTNSHVMQYGDMSHK
QEFLFAYMGADLSNRSHTSTSDISSPSISRAVDQRDTKLLYFQQKLQRAPTGSQEKQGAQK
QLLLEIAHRKNVDYSITKLGEALFGHEKSSNVLMNVRPQGQPVVDNWDCFKNFLNIYEK
YCGHLSAYGMKYTRAIANICNAGITTEKMVAASDQTCANKPNV

>MDP0000937205 Malusdomestica

MACGALLFVALLSLIVGSWCLPENAELKDFGTNKDNGPPSNTEKKGNRWAVLVAGSNEY
YNYRHQADISHAYQILKKGGLKDENIIVFMYDDIAYNPENPRQGVIVNKPNGPNVYKGVP
KDYTGTDVNSNNFYNVILGNKSALTGGSGKVLLSGPNDHVFIYYADHGSAGLLAMPTDG
DYVYAKDLIRVLKKKHASRGFKSMVLYVEACESGSMFDGILPRNLNIYATTSANPEESSYG
TYCPGEDPPVAQEYGTCLGDLYSISWMEDCDISDSRKETLQQQYERVRRRTNKSHVMQY
GDMSRRQQFLVTYMGGYLSKHSTSTSDISSPLISRVVNQRDTKLHYFQHKLRRAPTGSQE
ESEARKQLLDEIAHRKHVDDSIHKIGELLFGHRESSNMLMKGRPRGQPLVDNWDCFKKLL
KIYEKYCGHFSAYGMKHTRAIANMCNAGITAEKMVVASDQTCSKKPHV

>MDP0000188488 Malusdomestica
MRTFLILITILLSLSCESVANGFKLMPSEFPEDFIIHVNTASSSSSTVADQQNGTRWAVLVAGS
FGYGNYRHQADVCHAYQILKKGGLKDENIIVFMYDDIANNVENPRPGVIINKPDGPDVYK
GVPKDYTGENVTAANLYAVILGNKTALSGGSGKILDSGPNDNVFIYYTDHGASGIIGMPEG
DYVYANDLVDVLKQKHEAKGYKKMVFYLESCESGSMFEGLLPSNISIYATTAANATQDSW
ATYCPGFDPAPPAGYDTCLGDLYSISWMEDCDVKDLRRETFANQYETVRNRTANTSEGLG
SHVTQYGNLNQAKDLLFSYLGSDDNSTTYTNTKSSPSFLGPVFNQRDADLLHFLHKVRK
APDGSHEKHEAKQKLSAEMYRRERIDDNINQIAQLVFRXDSSSKMKNIRRNGQALVDDW
NCFKTFVKTYEKYCGALSNYGMQYTRIIANMCNYGVTMEQMTAASTKTCS

>Sme2.5_00297.1_g00008.1 Solanum melongena
MLLVMVGAMSFEPKIGRRLGRPHRLWDPLIRSPVDRDDDETEEGGGGVRWAVLVAGSNG
YGNYRHQADVCHAYQILKRGGLKDENIVVFMYDDIAKSELNPRPGIIINHPNGSDVYAGV
PKDYTGEHVTAANLYAVLLGDKGAVKGGTGKVVNSGPNDRIFLYYSDHGGPGVLGMPN
MPYVYGKDLIEVLKKKHAAGTYKEMVLYIEACESGSVFEGLMPENLNIYVTTASNAEESS
WGTYCPGMDPPPPSEYITCLGDLYSVAWMEDSESHNLKKETIKQQYEKVKERTSNFKNYE
AGSHVMEYGSKDIKPEKVYLYQGFDPATVNLPANKIDFARLEVVNQRDADLLFLWERYK
KLEDNSFEKANLRKEIAETMRHRQHLDGSIDAVGVFLFGPIKGSSVLNSVRKPGLPLVDD
WECLKSTVRLFEAHCGSLTQYGMKHMRAFANICNNGISRDAMEEAFMAACNGHSIEEHS
AANRGVSAS

>Sme2.5_03772.1_g00001.1 Solanum melongena
MKIHVAGVFIFVGLSIFVAVEGRNVLKLPSETSRFFDDDADDSIGTRWAVLLAGSNNYWNY
RHQADVCHAYQLLRKGGLKDENIIVFMYDDIAYNEENPRPGVIINSPAGEDVYKGVPKDY
TGGDVNVNNFLAVLRGDKTALTGGSGKVVNSGPDDHIFIFYSDHGGPGVLGMPTYPYLYA
KDLIDVLEKKHAAGTYKSMVLYIEACESGSIFEGLLPKGLNIYATTASNAEESSWGTYCPG
EYPSPPPEYETCLGDLYAVSWMEDSEMHNLRTENLRQQYHLVKRRTANGNSAYGSHVMQ
FGDLQLSMESLFRFMGTNPANDNYTFVDDNSLWASSEAVNQRDADLLHFWDKFRKAPEG
SARKVEAQKQFTEAMSHRMHLDNSIALVGKLLFGIQKGPEVLKHVRSAGQPLVDDWACL
KSFVRTFESHCGSLSQYGMKHMRSIANICNAGIKMEQVVEASAQACPTVPSNTWSSLHRG
FSA

>Sme2.5_03329.1_g00006.1 Solanum melongena
MNGYVAGILFLIGFAAVSESRNFLKLPSEGSDDSVGTRWAILLAGSNGYWNYRHQADICH
AYQLLKKGGLKDENIVVFMYDDIANSEENPRPGVIINSPHGEDVYRGVPKDYTGDDVTV

NNFLAALLGNKTALTGGSGKVVDSGPDDHIFIFYSDHGGAGVLGMPTNPYLYANDLVAAL
KKKHASGTYKSLVFYLEACESGSMFEGLLPEGLNIYATTASNADESSWGTYCPGEYPSPPI
EYETCLGDLYSISWMEDSERHNLRTESLKQQYHLVKERTAHGNPTYGSHVMQYGDVHLS
KNALFLYMGTDPANDNYTFMDDNSLQISKAVNQRDADLVHFWYKFHKAPEGSVRKTEA
QKQLNEAISHRMHLDISIALVGKLLFGIKKGPEVLASVRPAGQPLVDNWDCLKSYVRTFET
YCGSLSQYGMKHMRSVANICNAGIKMEQMVEASAQACPSVPSNTWSSLHSGFSA

>Sme2.5_01180.1_g00010.1 Solanum melongena
MYLIKISIWAKIEGRSISRFLTEETVGTKWAVLVAGSNGWWNYRHQADVCHAYQLLKKGG
VKDENIIVFMYDDIANNTMNPRPGVIINNPHGQDVYKGVPKDYVGEDVNAENFFNVILA
NKSGITGGSGKVLNSGPNDHIFIYYTDHGGPGIVSMPTGVVYANDLIDVLKKKHASGTYS
KLVFYLEACESGSMFDGLLPEGLDIYVTTASNPNESSWGTYCGVGDARDPCLVECPPPEFK
GVCLGDLYSVAWMEDSDVHDRQTETLDDQYDRIANRTAANLTYGSHVMQYGDMALSV
DALFQYMGAASTNHTHVSMNSKSSSQNVDQRDTELFYWQSKYEDAPKGSGEYFEAGAR
LIKVVAHRSQVDNSVKHIGELLFGVDFGSEVLQNVRPAGQPLVDNWDCLKSYVEKFEAH
CGKLSSYGKKHIRGIANICNAGIESEQIDAAIEQACGPLYS

>Sotub08g025020.1.1 Solanum tuberosum
MGSCNFTVCVTLMLLVMVGAISFEPKIDTRIGRAHRLWDPLIRSPVDRDDDEMEEDGGGV
RWAVLVAGSNGYGNYRHQADVCHAYQILKRGGLKDENIVVFMYDDIAKSELNPRPGVIIN
HPNGSDVYAGVPKDYTGEHVTAANLYAVLLGDKSAVKGGSGKVVNSGPNDRIFLYYSDH
GGPGVLGMPNMPYLYGKDLIEVLKKKYAAGTYKEMVLYIEACESGSVFEGLMPENLNIY
VTTASNAEESSWGTYCPGMDPPPPSEYITCLGDLYSVAWMEDSESHNLKKETIKQQYEKV
KERTSNSNNYNAGSHVMEYGSKEIKPEKVYLYQGFDPATVNLPANKIDFARLEVVNQRDA
DLLFLWERYKKLEDNSLEKAKLRKEITETLQHRQHLDGSIDAVGVFLFGPIKGGSVLSSVR
KPGLPLVDDWECLKSTVRLFEAHCGSLAQYGMKHMRAFANICNNGISRDAMEEAFMAA
CNGHTEEYSAANRGFSA

>Sotub08g015300.1.1 Solanum tuberosum
MNRSIAGVLFLIALSLNVSVSESRNFLKLPSEGSRFFDADENDSVGTRWAILLAGSNGYWN
YRHQADICHAYQLLKKGGLKDENIVVFMYDDIANNEENPRQGVIINSPHGEDVYKGVPK
DYTGDDVTVNNFLAALLGNKTAITGGSGKVVDSGPNDHIFIFYSDHGGAGVIGMPTDPYL
YANDLIDALKKKHASGTYKSLVFYLEACESGSMFEGLLPEGLNIYATTASNADESSWGTY
CPGEFPSPPIEYGTCLGDLYSISWMEDSERHNLRTETLKQQYHLVKERTASGNPAYGSHVM
QYGDVHLSKDVLFLYMGTDPANDNSTFMDDNSMRVSKAVNQRDADLVHFWYKFHKAP
EGSVRKTEAQKQLNEAISHRMHLDNSIALVGKLLFGIKKGPEVLTSVRPAGQPLVDDWDC
LKSYVRTFETHCGSLSQYGMKHMRSVANICNAGIKMEQMVEASAQACPSVPSNTWSSLQ
RGFSA

>Sotub12g028270.1.1 Solanum tuberosum
MIHVAGVFILVGVAVLAAVEGRNVLKLPSEASRFFDVADDSVGTRWAVLLAGSNGYWNY
RHQADVCHAYQLLRKGGLKDENIIVFMYDDIAHHEENPRPGVIINSPAGEDVYEGVPKDY
TGDDVNVHNFLTVLLGNKTALTGGSGKVVNSGPNDHIFIFYSDHGGPGVLGMPTNPYLYA
NDLIAVLKKKHAAGTYKSLVLYIEACESGSIFEGLLPKGLNIYATTASNAEESSWGTYCPGE

YPSPPPEYETCLGDLYAVSWMEDSEMHNLRTENLRQQYHLVKKRTANGNTAYGSHVMQF
GDLQLSMESLFRFMGTNPANDNYTYVDDNSLWASSKAVNQRDADLLHFWDKFRKAPEG
SARKVEAQKQFTEAMSHRMHLDERIALVGKLLFGIQKGPEVLKHVRSAGQPLVDDWACL
KSFVRTFESHCGSLSQYGMKHMRSIANICNAGVQMEQMVEASVQACPSIPSNTWSSLHR
GFSA

>Os04g0537900 Oryza sativa
MAARCWVWGFVVALLAVAAAADGEEEEGKWEPLIRMPTEEGDDAEAAAPAPAPAAADY
GGTRWAVLVAGSSGYGNYRHQADVCHAYQILQKGGVKEENIVVFMYDDIAHNILNPRPG
TIINHPKGGDVYAGVPKDYTGHQVTTENFFAVLLGNKTAVTGGSGKVIDSKPEDHIFIYYS
DHGGPGVLGMPNLPYLYAGDFIKVLQKKHASNSYSKMVIYVEACESGSIFEGLMPENLNI
YVTTASNAVENSWGTYCPGEEPSPPPEYITCLGDMYSVAWMEDSETHNLKKETIEDQYEL
VKKRTSNANKLNEGSHVMEYGDKTFKDEKLFLYQGFNPANGNITNELIWPVPKATVNQR
DADLLFMWKRYEQLNGVSEDKLRALREIEDTIAHRKHLDSSIDFIGKLVFGFENGPLALEA
ARSSGQPLVDNWDCLKKMVRIFESQCGSLTQYGMKYMRAFANICNNGVSEAKMMEASI
NACGRYNSARWSPMTEGGHSA

>Os01g0559600 Oryza sativa
MAARARLRLVLPPLAALLLFAHLAAVAVARPRWEEEGSNLRLPSERAVAAGAAADDAAEA
AEGTRWAVLIAGSNGYYNYRHQADVCHAYQIMKRGGLKDENIIVFMYDDIAHNPENPRP
GVIINHPQGGDVYAGVPKDYTGKEVNVKNLFAVLLGNKTAVKGGSGKVLDSGPNDHIFIF
YSDHGGPGVLGMPTYPYLYGDDLVDVLKKKHAAGTYKSLVFYLEACESGSIFEGLLPNGI
NVYATTASNADESSWGTYCPGEYPSPPPEYDTCLGDLYSVAWMEDSDVHNLRTESLKQQ
YNLVKERTSVQHTYYSGSHVMEYGSLELNAHHVFMYMGSNPANDNATFVEDNSLPSFSR
AVNQRDADLVYFWQKYRKLPESSPEKNEARKQLLEMMAHRSHVDNSVELIGNLLFGSEE
GPRVLKAVRATGEPLVDDWSCLKSMVRTFEAQCGSLAQYGMKHMRSFANICNAGISAEA
MAKVAAQACTSIPSNPWSSTHRGFSA

>Os02g0644000 Oryza sativa
MAARWCFALLLALSAAAAGAGAKRTWEPVIRMPGEVVEEEVATVPRGSEGTEEEEKDGV
GTRWAVLVAGSSGYGNYRHQADVCHAYQILRKGGLKEENIVVFMYDDIANNILNPRPGVI
VNHPQGEDVYAGVPKDYTGDEVTAKNFYAVLLGNKTAVTGGSRKVIDSKPNDHIFIFYSD
HGGPGVLGMPNLPYLYAADFMKVLQEKHASNTYAKMVIYVEACESGSIFEGLMPEDLNI
YVTTASNAEESSWGTYCPGMEPSPPSEYITCLGDLYSVSWMEDSETHNLKEESIKKQYEV
VKKRTSDMNSYGAGSHVMEYGDRTFKDDKLYLYQGFDPANAEVKNKLSWEGPKAAVN
QRDADLLFLWRRYELLHDKSEEKLKALREISDTVMHRKLLDSSVDLVGKLLFGFGNGPSV
LQAVRPSGQPLVDDWDCLKRMVRIFESHCGPLTQYGMKHMRAFANICNNGISGASMKEA
SIATCSSHNSGRWSSLVQGYSA

>Os05g0593900 Oryza sativa
MGRGLLCLLLLQLVGLVVAGGGRWRWQEEFLRLPSSDETTRWAVLIAGSNGFYNYRHQA
DVCHAYQIMRKGGVEEQNIVVMMYDDIAHNPDNPRPGLIFNHPSGPDVYAGVPKDYTGD
DVNVNNFLAVLLGNRSALTGSGSGKVVASGPNDHVFVYYADHGGPGVLSMPADGEYLYA
DDLVKALKKKHAGGGYKSLVVYVEACESGSIFEGLLPSDISVATTASNAEESSWGTYCPG

DDHDAPAAEFDTCLGDLYSVAWMEDAEAHQEGRLAETLRQQYRTVKNRTSDEGTYTLGS
HVMQYGDMALAPQSLDLYYMDTSPATANDHKLAAAGAKGSHSYTVSVNQRDADLLYL
WRKYRRAGEGTAEKVEARERLVQEMGRRSRVDRSVEMIGGLLLGGAKHKQQVVRERAA
LVEDWECLRSMVRTFEDQCGSLGQYGIKHMRSFANICNAGVPHHAMAKAASLACPSPPP
LHL

>IbVPE1
MIRSVVASLLLLTVSIVAVADGRGFLKLPSEARRFFRPAEEENREADGDDSVGTRWAVLIAG
SNGYWNYRHQADICHAYQILKAGGLKDENIVVFMYDDIAYNEENPRKGIIINSPHGEDVY
HGVPKDYTGDDVTVNNLLAVILGDKSAVKGGSGKVVDSGPNDHIFIYYSDHGGPGVLGM
PTSPYLYADELNAALKKKHAAGAYKSLVFYLEACESGSIFEGILPKDINIYATTASNAIESSW
GTYCPGEYPSPPPEYETCLGDLYSIAWMEDSDIHNLRTESLKQQYNLVKDRTLNGNTAYGS
HVMQYGDLELNADSLFMYMGTNPANENFTFVDEKSLKLSAPRRAVNQRDADLLHFWDK
FRNAPEGSARKSEAQKQFTEAITHRTHLDNSIALVGKLLFGIEKGPEVLSSVRATGLPLVDD
WSCLKSYVRAFETHCGSLSQYGMKHMRSIANICNAGISEERMAEASAQACPTFPSYSWSS
LRGGFSA

FigS4: quantitative analyses of leaf traits in WT and OX plants.; (a) petiole length; (b) blade length; (c) blade width; (d) blade perimeter; (e) blade area; (f) number of rosette leaves; (g) analysis of flowering times in WT and OX plants; asterisks (＊) indicate significant differences; t-test, $p < 0.05$.

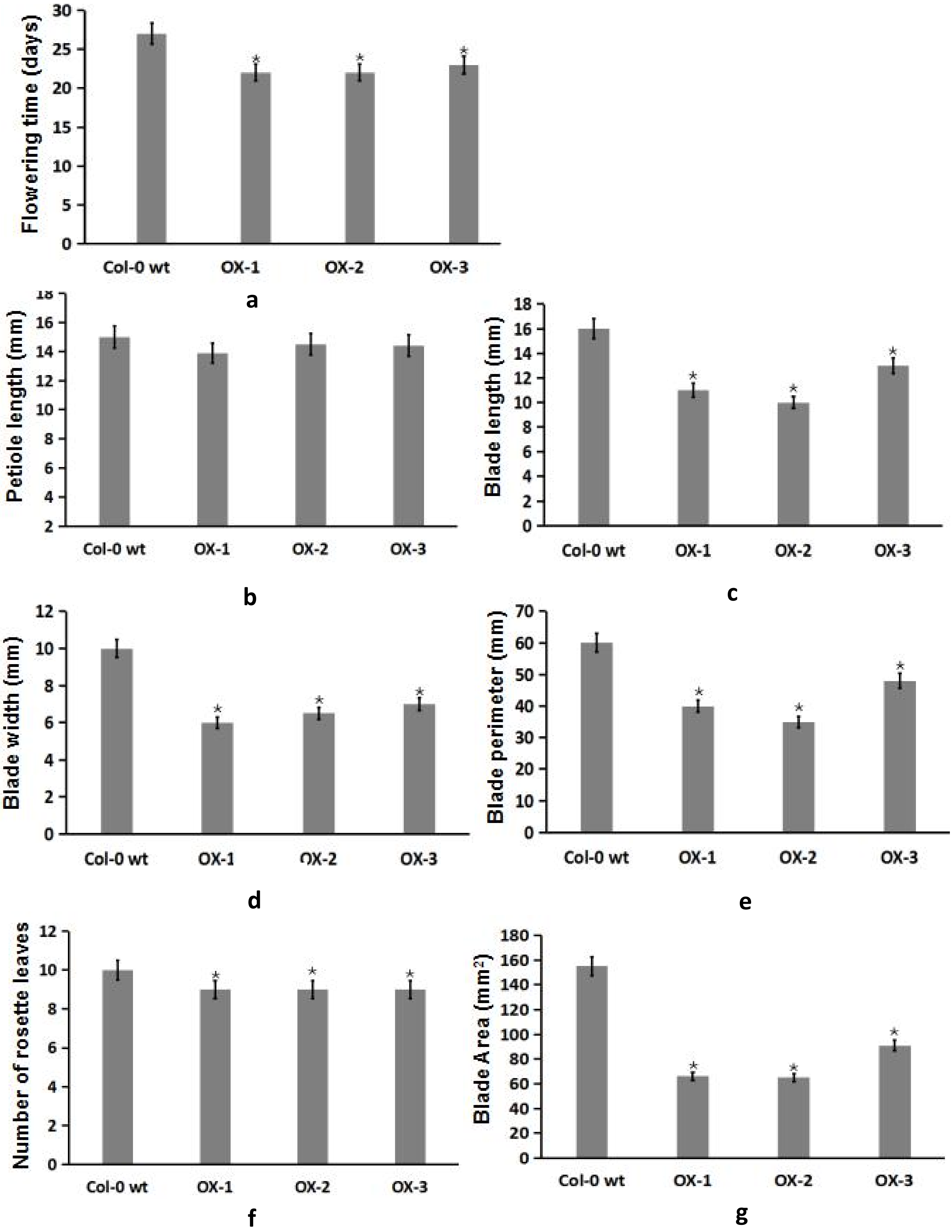
Flowering time (days)
0
5
10
15
20
25
30
Col-0 wt
OX-1
OX-2
OX-3
*
a
Petiole length (mm)
2
4
6
8
10
12
14
16
18
Col-0 wt
OX-1
OX-2
OX-3
b
Blade length (mm)
0
2
4
6
8
10
12
14
16
18
Col-0 wt
OX-1
OX-2
OX-3
*
c
Blade width (mm)
0
2
4
6
8
10
12
Col-0 wt
OX-1
OX-2
OX-3
*
d
Blade perimeter (mm)
0
10
20
30
40
50
60
70
Col-0 wt
OX-1
OX-2
OX-3
*
e
Number of rosette leaves
0
2
4
6
8
10
12
Col-0 wt
OX-1
OX-2
OX-3
*
f
Blade Area (mm2)
0
20
40
60
80
100
120
140
160
180
Col-0 wt
OX-1
OX-2
OX-3
*
g
