# Supplementary material for: Expression of IbVPE1 from sweet potato in Arabidopsis affects leaf development, flowering time and chlorophyll catabolism
Source: BMC Plant Biol. 2019 May 6;19:184. doi: 10.1186/s12870-019-1789-8 (PMC6503384; doi:10.1186/s12870-019-1789-8)
Supplement: Supplementary file 2 — Table S1. Gene-specific primers used in full-length cDNA and RT-PCR analysis (PDF 80 kb) [file 12870_2019_1789_MOESM2_ESM.pdf]

Table S1. Gene-specific primers used in full-length cDNA and RT-PCR analysis

Gene	Primers (5' to 3')	
IbVPE1 Full- length	**Forward**	ATGATTCGCTCCGTCGTC
	**Reverse**	TTATGCACTGAATCCTCCACG
IbVPE1 RT-PCR	**Forward**	CTCCAGAAGGCTCAGCAAGG
	**Reverse**	AGTAGCACGGACGCTGTTCA
IbVPE1 Promoter	**Forward**	GGTTTCCAAGAACCCCCTTTAGGT
	**Reverse**	CTAAAGGGGGTTCTTGGAAACCTT
Ib Ttublin	**Forward**	CAACTACCAGCCACCAACTGT
	**Reverse**	CAGATCCTCACGAGCTTCAC
TCP7	**Forward**	CAACAACAACAACAATAACAACGATGG
	**Reverse**	AGCAAAGTCGATGGAAGAGGGAGAAG
TCP8	**Forward**	ATGGATCTCTCCGACATCCGAAACAACA
	**Reverse**	TCACTCAGAGCTATTTGAGTTCTCCTCT
TCP14	**Forward**	ATGCAAAAGCCAACATCAAGTATCTTAAAT
	**Reverse**	GATGGTTCAGCTTGTTGAAGAAGCCACTCT
TCP15	**Forward**	ATGGATCCGGATCCGGATCATAACCATC
	**Reverse**	CTAGGAATGATGACTGGTGCTTCCATCT
TCP21	**Forward**	ACAACGACGGAGCAGTGAG
	**Reverse**	GCGCAAATGATTGGCATA
TCP22	**Forward**	ATGAATCAGAATTCCTCTGTTGCGGAGG
	**Reverse**	TCACTTTTTGTCATCACCACCATTTTCA
TCP23	**Forward**	ATGGAGTCCCACAACAACAACCAGAGCA
	**Reverse**	TCAAGGAGAACCATCTATAGTAGGATTT
LFY	**Forward**	TTGTGGGTATGAAGGACGAGGAGC
	**Reverse**	TTCCTCCTCCGCCGTTATTCCC
AP1	**Forward**	TGCAAGCAATGAGCCCTAAAGAGC
	**Reverse**	GGAGATGGCTGATGAGAGAGCATG
SOC1	**Forward**	GGATCGAGTCAGCACCAAACCG
	**Reverse**	GGTAACCCAATGAACAATTGCGTC
CO	**Forward**	GCCATAAACGTGTCCGGGTCTG
	**Reverse**	ACAGCCACGAAGCAACCTCCTTG
FT	**Forward**	TTCCAAGTCCTAGCAACCCTCACC
	**Reverse**	TTCTTCCTCCGCAGCCACTCTC
FRI	**Forward**	CAGGAGATTGTACCGGAGACGTCG
	**Reverse**	CCTTCCCTTTACCACGATCAGGC
FLC	**Forward**	CTCCGGCGATAACCTGGTCAAG
	**Reverse**	TCAGCTTCTGCTCCCACATGATG

**Gene**	**Primers (5’ to 3’)**	
BN1	**Forward**	TGACCGTTACTCCATTCGCATTGG
	**Reverse**	GTTGTGAGCGGAATCCATGAGACC
BN2	**Forward**	ATGGAATTGCCCTGCTGAATGATG
	**Reverse**	CCAGAGAAGAGTGGGCTATGGCTG
FPA	**Forward**	TCCCGTCTGTTGTGCTCGTTGTG
	**Reverse**	TGGCAACGGCGTAATGTTTAGCG
FVE	**Forward**	GAAACCCAACCAAACCGTCATGC
	**Reverse**	AAAGGGTTCCGTTGGGCACATG
FCA	**Forward**	GGCAATGGCAGCTATCGATGGTC
	**Reverse**	GGTGCCATTTCCCTTGACTCGC
LD	**Forward**	TGGACGCGTTCAAGGAGGAGATAG
	**Reverse**	CGCAACGACGACATCTTGGAGC
AtTubulin	**Forward**	CTCAAGAGGTTCTCAGCAGTA
	**Reverse**	TCACCTTCTTCATCCGCAGTT
NPT II	**Forward**	GACAATCGGCTGCTCTGA
	**Reverse**	AACTCCAGCATGAGATCC
